# Metastasis-associated *MCL1* and *P16* copy number alterations dictate resistance to vemurafenib in a *BRAF^V600E^* patient-derived papillary thyroid carcinoma preclinical model

**DOI:** 10.18632/oncotarget.6442

**Published:** 2015-11-30

**Authors:** Mark Duquette, Peter M. Sadow, Amjad Husain, Jennifer N. Sims, Zeus A. Antonello, Andrew H. Fischer, Chen Song, Elena Castellanos-Rizaldos, G. Mike Makrigiorgos, Junichi Kurebayashi, Vania Nose, Paul Van Hummelen, Roderick T. Bronson, Michelle Vinco, Thomas J. Giordano, Dora Dias-Santagata, Pier Paolo Pandolfi, Carmelo Nucera

**Affiliations:** ^1^ Laboratory of Human Thyroid Cancers Preclinical and Translational Research, Division of Cancer Biology and Angiogenesis, Cancer Research Institute (CRI), Cancer Center, Department of Pathology, Beth Israel Deaconess Medical Center, Harvard Medical School, Boston, MA, USA; ^2^ Department of Pathology, Massachusetts General Hospital, Harvard Medical School, MA, Boston, USA; ^3^ Department of Pathology, Center for Vascular Biology Research (CVBR), Beth Israel Deaconess Medical Center, Harvard Medical School, Boston, MA, USA; ^4^ Department of Pathology, University of Massachusetts, Worcester, MA, USA; ^5^ Department of Radiation Oncology, Dana Farber Cancer Institute, Harvard Medical School, Boston, MA, USA; ^6^ Department of Breast and Thyroid Surgery, Kawasaki Medical School, Kurashiki, Japan; ^7^ Center for Cancer Genome Discovery (CCGD), Dana Farber Cancer Institute, Harvard Medical School, Boston, MA, USA; ^8^ Rodent Histopathology Unit, Department of Microbiology and Immunobiology, Harvard Medical School, Boston, MA, USA; ^9^ Department of Pathology, University of Michigan, Ann Harbor, MI, USA; ^10^ Center for Integrated Diagnostics, Department of Pathology, Massachusetts General Hospital, Harvard Medical School, Boston, MA, USA; ^11^ Division of Cancer Genetics, Cancer Research Institute (CRI), Department of Medicine and Department of Pathology, Cancer Center, Beth Israel Deaconess Medical Center, Harvard Medical School, Boston, MA, USA

**Keywords:** BRAF^V600E^ papillary thyroid cancer pre-clinical model, vemurafenib resisatnce, MCL1 and P16/CDKN2A somatic copy number, microenvironment

## Abstract

*BRAF^V600E^* mutation exerts an essential oncogenic function in many tumors, including papillary thyroid carcinoma (PTC). Although BRAF^V600E^ inhibitors are available, lack of response has been frequently observed. To study the mechanism underlying intrinsic resistance to the mutant BRAF^V600E^ selective inhibitor vemurafenib, we established short-term primary cell cultures of human metastatic/recurrent *BRAF^V600E^*-PTC, intrathyroidal *BRAF^V600E^*-PTC, and normal thyroid (NT). We also generated an early intervention model of human *BRAF^V600E^*-PTC orthotopic mouse. We find that metastatic *BRAF^V600E^*-PTC cells elicit paracrine-signaling which trigger migration of pericytes, blood endothelial cells and lymphatic endothelial cells as compared to *BRAF^WT^*-PTC cells, and show a higher rate of invasion. We further show that vemurafenib therapy significantly suppresses these aberrant functions in non-metastatic *BRAF^V600E^*-PTC cells but lesser in metastatic *BRAF^V600E^*-PTC cells as compared to vehicle treatment. These results concur with similar folds of down-regulation of tumor microenvironment–associated pro-metastatic molecules, with no effects in *BRAF^WT^*-PTC and NT cells. Our early intervention preclinical trial shows that vemurafenib delays tumor growth in the orthotopic *BRAF^WT/V600E^*-PTC mice. Importantly, we identify high copy number gain of *MCL1* (chromosome 1q) and loss of *CDKN2A* (*P16*, chromosome 9p) in metastatic *BRAF^V600E^*-PTC cells which are associated with resistance to vemurafenib treatment. Critically, we demonstrate that combined vemurafenib therapy with BCL2/MCL1 inhibitor increases metastatic *BRAF^V600E^*-PTC cell death and ameliorates response to vemurafenib treatment as compared to single agent treatment. In conclusion, short-term PTC and NT cultures offer a predictive model for evaluating therapeutic response in patients with PTC. Our PTC pre-clinical model suggests that combined targeted therapy might be an important therapeutic strategy for metastatic and refractory *BRAF^V600E^*-positive PTC.

## INTRODUCTION

The *BRAF^V600E^* mutation is the most common genetic alteration in papillary thyroid carcinoma (PTC) and may be associated with progression of PTC to anaplastic thyroid carcinoma (ATC) [1, 2], one of the most lethal human cancers [3, 4]. *BRAF^V600E^* is present in about 61% of PTCs as recently confirmed by the PTC TCGA (The Cancer Genome Atlas) and can be considered to be the primary genetic hallmark of PTC [5]. PTC patients harboring *BRAF^V600E^* mutation show resistance to radioiodine treatment [4, 6] [7], and have higher rates of recurrence and metastases, and lower survival rates [8-12]. Clearly, new therapeutic options are needed for metastatic and radioiodine-resistant thyroid cancers like *BRAF^V600E^*-positive PTC or ATC [13, 14]. Vemurafenib is the first orally available selective inhibitor of BRAF^V600E^ approved by the FDA (Food and Drug Administration) for the treatment of *BRAF^V600E^*-melanoma [15, 16]. Although vemurafenib has recently shown promising clinical activity in three patients with metastatic PTC [17], its lack of response with resistance has been frequently observed [18] [19]. To address the unmet clinical need in *BRAF^V600E^*-positive metastatic PTC, we have studied the potential mechanism of loss of responsiveness to the BRAF^V600E^ inhibitor vemurafenib, using a strategy referred to as a pre-clinical trial/model. We established short-term primary cell cultures of human thyroid samples, including *BRAF^V600E^*-PTC samples derived from metastatic/recurrent PTC, intrathyroidal primary PTC, and human normal thyroid (NT). Here, we describe the development of the first patient-derived *BRAF^WT/V600E^*-PTC *in vitro* and *in vivo* early intervention pre-clinical model with some similar disease molecular characteristics that are recapitulated. More importantly, this model offers interpretative insight into the concurrent vemurafenib human clinical trials in an independent cohort of patients with metastatic *BRAF^V600E^*-PTC, may serve to provide rapid clinical translation of our findings. We aim to investigate somatic copy number alterations (SCNAs) which could be associated with metastatic *BRAF^WT/V600E^*-PTC and mechanistically render these carcinomas resistant to the effects of *BRAF^WT/V600E^* inhibitors (e.g. vemurafenib) on cell death. We identify high copy number gain of *MCL1* (myeloid cell leukemia sequence 1, chromosome 1q) and loss of *CDKN2A (P16*, chromosome 9p) in metastatic *BRAF^V600E^*-PTC cells which are associated with intrinsic resistance to vemurafenib treatment. Collectively, our PTC pre-clinical model suggests that combination of anti-*BRAF^WT/V600E^* therapy (e.g. vemurafenib) with inhibitors of pro-survival molecules (i.e. pan-BCL2/MCL1 inhibitors) ameliorates intrinsic resistance to metastatic *BRAF^V600E^*-PTC cell death.

## RESULTS

### Vemurafenib therapy impairs viability of human non metastatic PTC cells in a pre-clinical model of patient-derived PTC harboring BRAF^WT/V600E^

**Figure 1:**
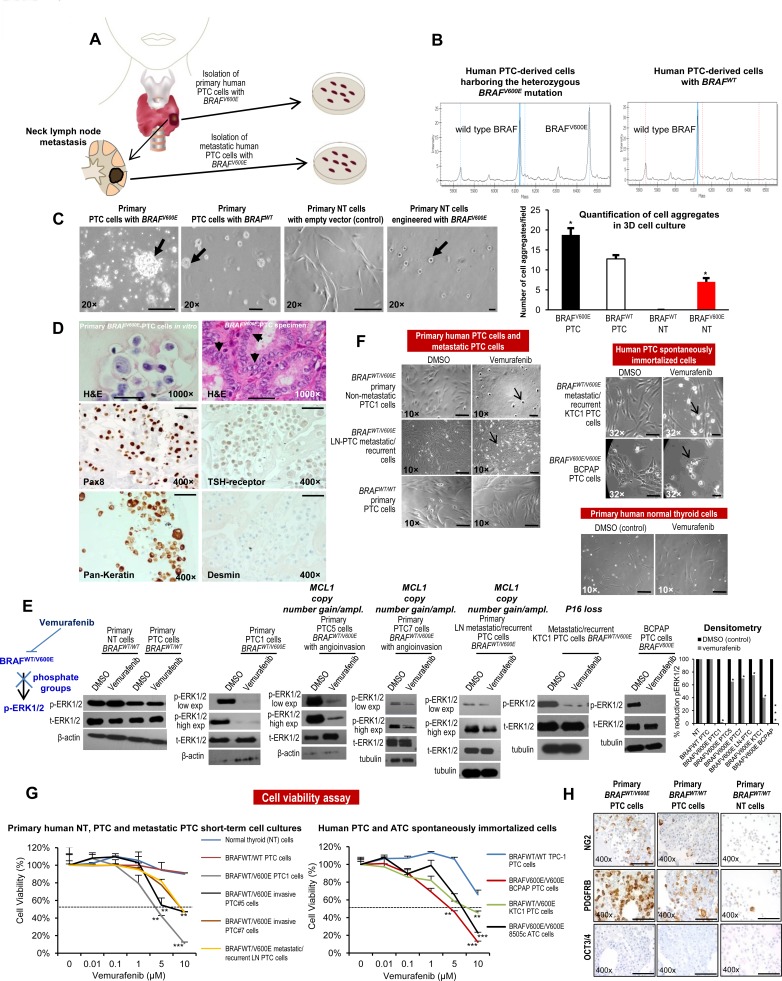
*In vitro* preclinical model of human papillary thyroid cancer (PTC) harboring the BRAF^V600E^ mutation **A**. Experimental design of an *in vitro* and *in vivo* model of human PTC with the *BRAF^WT/V600E^* mutation. **B**. DNA genotyping analysis of human PTC identifies the heterozygous *BRAF^WT/V600E^* mutation. Mass spectrometry (MS) traces of human primary PTC cells. The intensity of the signal versus mass of the analyte is plotted in the background. Calls are based on an expected allelic frequency of 50%. Allele frequencies deviating from the expected values are assigned ambiguous or homozygous calls by the software. MS trace of PTC cells reveals a heterozygous BRAF^WT/V600E^ allele (A>T). **C**. In a three dimensional (3D) cell culture assay using reconstituted basement membrane extracellular matrix (ECM) (Matrigel), *BRAF^V600E^*-PTC cells grew as larger cell aggregates. Normal thyroid (NT) cells transduced with *BRAF^V600E^* grew as adherent refractile cells vs. NT cells engineered with empty vector (control) which grew as spindled cells. Scale bar= 400 μ, 200 μ, 400 μ and 50 μ, respectively. **D**. Immunocytochemistry of representative established short-term primary human PTC cells *in vitro* with the heterozygous *BRAF^WT/V600E^* mutation of patient-PTC specimen (Hematoxylin-Eosin, H&E, arrows highlight nuclear clearing). Immunocytochemistry staining in the PTC cells *in vitro* shows cytoplasmic to membranous staining with antibodies against PAX8, TSH-receptor, and pan-keratin (marker of tumor epithelial cells and tumor purity). Desmin immunostain was negative. Scale bars= 500 μ (1000× magnification image) and 100 μ (400× magnification images). **E**. Inhibition of BRAF^WT/V600E^ by vemurafenib reduces phospho(p)ERK1/2 protein expression levels. A parallel plate similar to F was set up and corresponding pERK1/2 protein levels (low exp= shorter exposure during chemiluminescence reaction; high exp= longer exposure during chemiluminescence reaction) were measured from *BRAF^WT/V600E^*-PTC cells, *BRAF^WT^*-PTC cells, or NT cells by western blotting. Densitometry analysis of the pERK1/2 protein levels in NT or PTC cells treated with 10 μM vemurafenib vs. vehicle (DMSO =Dimethyl sulfoxide, control) for 24 hours, in the corresponding western blotting (*p < 0.05, Mann-Whitney test). Primary *BRAF^WT^*-NT cells have *MCL1* neutral copy number, primary *BRAF^WT^*-PTC cells have *MCL1* copy number =0.9, primary non-metastatic *BRAF^WT/V600E^*-PTC1 cells have *MCL1* copy number =2.14, primary *BRAF^WT/V600E^*-PTC5 cells with angio-invasion have *MCL1* copy number =3, primary *BRAF^WT/V600E^*-PTC7 cells with angio-invasion have *MCL1* copy number =3, primary LN metastatic/recurrent *BRAF^V600E^*-PTC cells have *MCL1* copy number =3.8, KTC1 cells have *MCL1* copy number =1.3 and BCPAP cells have *MCL1* copy number =1.4. KTC1 cells have *P16* homozygous loss. For more details regarding copy number gain/amplification (ampl.) assay see Figure 4 and Methods. These data are representative of three independent experiments. We show these results in the 5 out of 7 short-term primary human PTC cell cultures which grew well. **F**. Arrows highlight change of cell shape in *BRAF^WT/V600E^*-PTC cells treated with vemurafenib vs. vehicle-treated (control) PTC cells. PTC cells with heterozygous *BRAF^WT/V600E^* or *BRAF^WT^* or NT cells were treated with 10 μM of vemurafenib or with DMSO (control) for about 24 hours. These data represent 3 independent experiments. All scale bars are=50 μ (DMSO images) and 10 μ (Vemurafenib images). Scale bars are =50 μ (BRAF^WT/WT^ primary PTC cells and primary human normal thyroid cells images). **G**. Vemurafenib dose-reponse analysis: short-term primary human PTC or NT cells with *BRAF^V600E^* or with *BRAF^WT^*, as well as spontaneously immortalized human PTC and ATC cells, were treated with the indicated concentrations of vemurafenib for 48 hours, and viability was determined using the Cell Titer-Glo ATP-based luminescence assay, with DMSO-treated cells as the control. We show these results in the 5 out of 7 short-term primary human PTC cell cultures which grew well. These data represent the average ± standard deviation (error bars) of 3-5 independent replicate measurements (*p < 0.05, **p < 0.01, ***p < 0.001, Mann-Whitney test). **H**. Immunocytochemistry of representative established non-immortalized primary human PTC cells with the heterozygous BRAF^WT/V600E^ mutation or with *BRAF^WT^*, or NT cells. Immunohistochemistry staining shows cytoplasmic to membranous staining with antibodies against NG2 or PDGFRB (platelet-derived growth factor receptor-beta) in BRAF^WT/V600E^-PTC or BRAF^WT^-PTC cells. OCT3/4 immunostain was negative. Markers expression was assessed semiquantitatively using the following scoring method: 0 (negative), 1+ (1–10% positive cells), 2+ (11–50% positive cells), and 3+ (more than 50% positive cells). All scale bars are=100 μ.

We have developed the first pre-clinical model of patient-derived PTC with *BRAF^WT/V600E^* (Figure 1A) using BRAF^WT/V600E^ inhibitors (i.e. vemurafenib). We established 7 short-term primary cell cultures *in vitro* of human PTC (which reduce the potential for changes *in vitro)*, including 2 from non angioinvasive and non-metastatic PTC, 2 from angioinvasive PTC with no clinical evidence of neck lymph node (LN) metastasis, 2 from angioinvasive PTC with positive clinical evidence of neck LN metastasis, and, 1 short-term cell culture from a PTC mediastinal LN metastasis (local metastasis) (Suppl. Table 1, Suppl. Figures 1A-B), all from individual patients with near-total thyroidectomy with primary PTC greater than 1.1 cm (pT1b-pT3). For comparison, we also established 4 independent cultures of primary normal thyroid (NT) cells from non-goiterous, non-thyroiditc tissue. All PTC samples were sequenced and showed wild type *TP53*. Overall, 71.4% (5/7) of the isolated PTC cell batches harbored the heterozygous *BRAF^WT/V600E^* mutation (Figure 1B). 14.2 % (1/7) harbored the *RET/PTC3* translocation without mutations in *BRAF* (Suppl. Figure 1E). No mutations included in our genomic sequencing panel were identified in 1 of the 7 PTC samples. Additionally, we have used KTC1 cells, a spontaneously immortalized *BRAF^WT/V600E^*cell line established from pleural effusion material (distant metastasis) from a male patient with metastatic and recurrent PTC with wild type *TP53* (weak nuclear expression, Suppl. Figure 2) which showed nuclear expression of PAX8 and phospho(p)-ERK1/2 proteins (Suppl. Figure 2). We also used BCPAP cells, with homozygous *BRAF^V600E^*, established from a poorly differentiated PTC. The NT cells showed no mutations. Furthermore, in order to assess the morphological growth pattern of these cells, we established a three-dimensional (3D) cell-culture system with Matrigel (extracellular matrix (ECM) *in vitro*) (Figure 1C). Representative *BRAF^V600E^*-PTC cells primarily grew significantly (p=0.02) as numerous cell aggregates (Figure 1C). Furthermore, NT cells engineered to express mutant BRAF^V600E^ grew significantly (p=0.02) as refractile cells compared to NT cells engineered with empty vector (Figure 1C). Representative NT and PTC cells expressed some thyroid differentiation biomarkers and epithelial tumor marker as indicator of tumor purity, and were negative for desmin (mesenchymal marker) (Figure 1D).

We tested the effects of vemurafenib using a dose-response in representative PTC cells with or without *BRAF^WT/V600E^* and in NT cells. Ten μM vemurafenib was an effective dose to substantially block the *BRAF^WT/V600E^* pathway, specifically reducing pERK1/2 protein expression levels by 98% (IC_90_) in non-metastatic *BRAF^WT/V600E^*-PTC cells as compared to the same cells treated with vehicle (control) within 24 hours and with no substantial changes in pERK1/2 protein expression levels in either PTC or NT cells with *BRAF^WT^* (Figure 1E). *BRAF^WT/V600E^*-PTC cells morphology was also substantially affected with this latter dose within 24 hours as compared to the vehicle-treated cells (Figure 1F). By contrast, metastatic *BRAF^WT/V600E^*-PTC cells were substantially less responsive and more resistant (IC_90_ for pERK1/2 protein levels was about 30%) to vemurafenib treatment within 24 hours (Figure 1E). The IC_50_ value (dose-response) for cell viability was 5 μM for vemurafenib in representative non-metastatic *BRAF^WT/V600E^*-PTC cells at 48 hours *in vitro* (Figure 1G) and was significant (p=0.001) as compared to vehicle-treated cells, with no effect on the viability of *BRAF^WT^*-PTC or NT cells (Figure 1G). In contrast, 10 μM vemurafenib was an effective (p=0.007) dose able to reduce viability of *BRAF^WT/V600E^*-positive metastatic PTC or ATC cells to about 50% vs. vehicle-treated cells (Figure 1G), with no significant effect on the viability of *BRAF^WT^*-PTC or NT cells (Figure 1G). Doses greater than 1 μM (e.g. 2 or 10 μM) have also been reported to be effective in *BRAF^V600E^*-melanoma or ATC cells *in vitro* [18] [20]. Moreover, because our primary PTC cells grew as cell aggregates (e.g. spheroids) in culture on the Matrigel, we also investigated the expression of stem-cell markers in PTC and NT cells *in vitro* (Figure 1H). Interestingly, we found that a sub-population of primary human *BRAF^WT/V600E^*-PTC cells expressed substantially about 2.5-fold more PDGFRB (tumor microenvironment-associated pro-angiogenic factor) protein vs. PTC cells with *BRAF^WT^* (Figure 1H).

### Effects of anti-BRAF^V600E^ therapy *in vivo*

**Figure 2:**
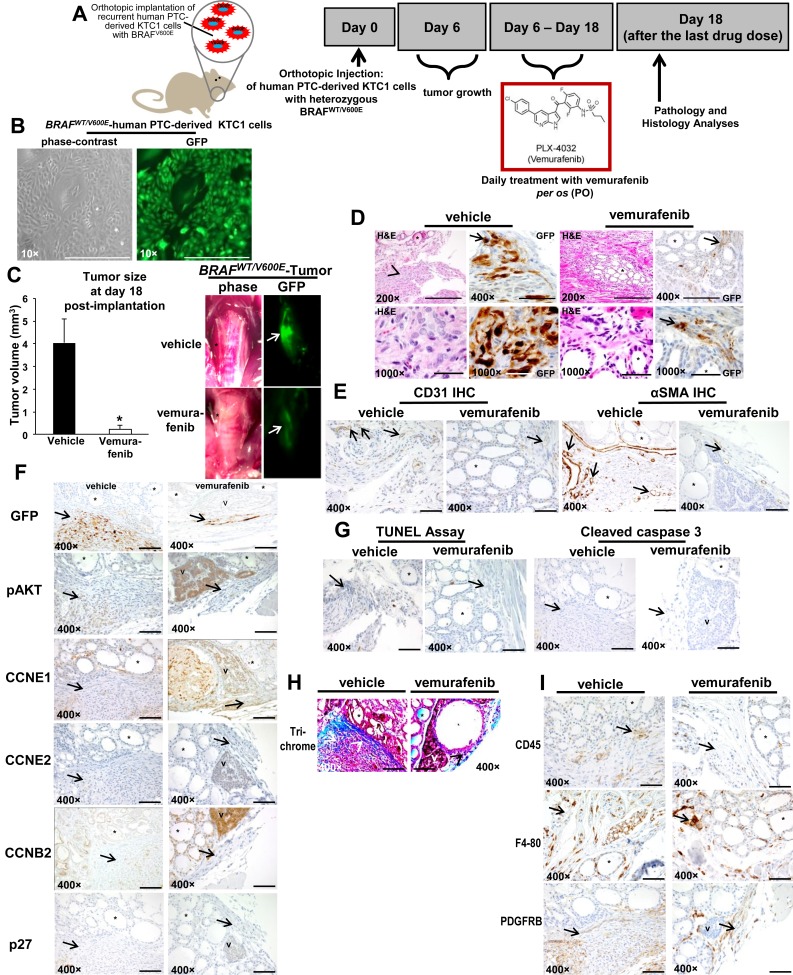
Early intervention model of an *in vivo* orthotopic mouse using KTC1 cells derived from a patient with papillary thyroid carcinoma (PTC) harboring the heterozygous BRAF^V600E^ mutation ***A***. Experimental design of an *in vivo* early intervention orthotopic preclinical model using KTC1 cells derived from a patient with PTC harboring the heterozygous *BRAF^WT/V600E^* mutation along with homozygous loss of *P16*. Nine weeks old male NOD SCID immunocompromised (gamma) (strain name: NOD.Cg-Prkdc^scid^ Il2rg^tm1Wjl^/SzJ) mice (n=10) were treated with vemurafenib or 2% solution of hydroxypropylcellulose (vehicle). Human KTC1 cells, engineered to express GFP were implanted in 10 mice. Their orthotopic tumors were evaluated by histology and green fluorescent protein (GFP) biomaging. Either vehicle (n=5) or vemurafenib (n=5) treatment was begun at 6 day post-tumor implantation, and the response to vemurafenib therapy was evaluated at 18 day. **B**. *In vitro* KTC1 cells engineered to express GFP. All scale bars are=400 μ.**C**. *BRAF^WT/V600E^*-KTC1 orthotopic tumor growth *in vivo* in the early intervention mouse model described in A. One week of vemurafenib treatment resulted in lower orthotopic tumor growth than in controls (vehicle treatment) (*p<005, Mann-Whitney test). Gross photo (contrast) shows in the right thyroid a small and non-palpable *BRAF^WT/V600E^* KTC1 orthotopic tumor (asterisk) (vehicle treatment) and a significant decrease of orthotopic tumor size with vemurafenib treatment (asterisk). This result was confirmed by GFP *ex-vivo* imaging (arrows) which shows a significant decrease of *BRAF^WT/V600E^* KTC1 orthotopic tumor in the vemurafenib-treated SCID mice vs. vehicle-treated mice. Scale bars= 400 μ (200× magnification image) and 500 μ (1000× magnification images). **D**. Vemurafenib inhibits *BRAF^WT/V600E^*-positive human KTC1 orthotopic tumor growth *in vivo* in the early intervention mouse model described in A. Control mice with human *BRAF^V600E^* KTC1 orthotopic tumor (H&E and GFP stain) showed tumor growth within a relatively circumscribed area, with mixed spindled and epithelioid features, and focal areas of nuclear clearing (H&E stain, vehicle). GFP stain highlights some of the more atypical tumor cells, with prominent, multifocal nuclear staining (arrow); vemurafenib-treated tumors (H&E and GFP stain) were small and discrete and confined to the thyroid bed (arrow highlights GFP+ tumor cells), sometime with hyper-chromatic nuclei. Arrows and arrowhead highlight orthotopic KTC1 tumor area, asterisk highlights mouse normal thyroid follicle. Abbreviations: H&E, hematoxylin and eosin. **E**. Microvascular density is reduced after vemurafenib treatment *in vivo* in the orthotopic mouse model of human KTC1 tumor with *BRAF^WT/V600E^* described in A. Vehicle-treated *BRAF^WT/V600E^* KTC1 orthotopic tumors had greater intratumoral staining for CD31 (arrows) (microvascular density) compared to vemurafenib-treated (control) mice (p<0.05, Mann-Whitney test). Microvascular density is defined by number of vessels per microscope field showing CD31 staining. Alpha Smooth Muscle Actin (αSMA, arrows) staining as highlighted with arrows indicates the significant reduction in vessel density in the vemurafenib-treated orthotopic tumors (2 vessels/field, arrows) vs. vehicle-treated orthotopic tumors (8 vessels/field, p<0.01). Asterisk highlights mouse normal thyroid follicle. All scale bars are=400 μ. **F**. Immunohistochemical protein expression of GFP, phospho(p)-AKT, cyclin E1 (CCNE1), cyclin E2 (CCNE2), cyclin B2 (CCNB2) and p27/kip1. At day 18, vehicle-treated (control) *BRAF^WT/V600E^* KTC1 orthotopic mice show strong GFP expression (arrow), which marks KTC1 tumor cells. In contrast, vemurafenib-treated *BRAF^WT/V600E^* KTC1 orthotopic mice show a smaller tumor as highlighted by GFP labeling (arrow). Vehicle-treated control *BRAF^WT/V600E^* KTC1 orthotopic tumors show weak and focal nuclear staining and peri-nuclear dot-like staining for pAKT (arrow) (scoring, 1+), nuclear and cytoplasmic staining for CCNE1 (arrow) (scoring, 1+), and nuclear and cytoplasmic staining for CCNB2 (arrow) (scoring, 1+) at day 18. *BRAF^WT/V600E^* KTC1 orthotopic tumors treated for 12 days with vemurafenib showed a decrease of pAKT, and CCNB2 (only peri-nuclear punctate staining, scoring 0) protein levels. No significant changes were observed in CCNE1, CCNE2 (arrow) (very low levels) or p27/kip1 (arrow) (very low levels) protein levels in the vehicle-treated BRAF^WT/V600E^ orthotopic tumors vs. those treated with vemurafenib. Asterisk highlights mouse normal thyroid follicle and V highlights mouse parathyroid. Immunohistochemical protein expression was assessed semi-quantitatively using the following scoring method: 0 (negative), 1+ (1–10% positive cells), 2+ (11–50% positive cells), and 3+ (more than 50% positive cells). **G**. Apoptosis assay was performed using terminal deoxynucleotidyl transferase dUTP nick end labeling (TUNEL) assay (peroxidase system) in *vivo* in the orthotopic mouse model of human KTC1 tumors with *BRAF^WT/V600E^* described in A. No significant difference was found in nuclear staining (apoptotic cells) in the vemurafenib-treated *BRAF^WT/V600E^* KTC1 orthotopic tumors at day 18 compared with vehicle (control)-treated tumors. Immunohistochemical protein expression of cleaved caspase 3: vehicle-treated control tumors (arrow highlights orthotopic tumor area) did not show cleaved caspase 3 protein expression at 18 day. Orthotopic tumors treated with 1 week of vemurafenib therapy do not show a significant increase of cytoplasmic cleaved caspase 3 protein levels at 18 day in the small tumor area (arrow). Asterisk highlights mouse normal thyroid follicle and V highlights mouse parathyroid. All scale bars are=400 μ. **H**. Trichrome staining highlights the substantial intratumoral deposition of collagen in the vehicle-treated *BRAF^WT/V600E^* KTC1 orthotopic tumors (blue staining, arrowheads) compared with lower levels of staining in the vemurafenib-treated tumors mice. Asterisk highlights mouse normal thyroid follicle and V highlights mouse parathyroid. All scale bars are=400 μ. **I**. Assessment of *BRAF^WT/V600E^* KTC1 orthotopic tumor microenvironment in the *in vivo* mouse model described in A. Immunohistochemical analysis of CD45 protein expression (arrow) (leucocyte marker), F4-80 (arrow) (macrophage marker) and PDGFRB (arrow) (platelet-derived growth factor receptor beta, pericyte lineage and tumor cells marker) was performed in orthotopic tumors from mice treated with vemurafenib or vehicle-only controls. CD45 and F4-80 was localized to plasma membrane and PDGFRB expression was localized in the cytoplasm/plasma membrane of spindle-shaped pericytes. No changes in CD45 and F4-80 protein levels in vehicle-treated mice vs. vemurafenib-treated mice were observed. PDGFRB protein expression levels were higher in the peri-tumor and intra-tumor areas in the vehicle-treated mice (score 3+) vs. vemurafenib-treated mice (score 1+). Asterisk highlight mouse normal thyroid and V highlights mouse parathyroid. Immunohistochemical protein expression was assessed semi-quantitatively using the following scoring method: 0 (negative), 1+ (1–10% positive cells), 2+ (11–50% positive cells), and 3+ (more than 50% positive cells). All scale bars are=400 μ.

We have developed the first early intervention pre-clinical mouse trial of human PTC-derived cells with *BRAF^WT/V600E^* using vemurafenib (Figure 2A-2B). Immunocompromised mice were orthotopically implanted with the human KTC1 cells derived from a metastatic/recurrent *BRAF^V600E^*-positive PTC. These cells were engineered to express green fluorescent protein (GFP). KTC1 orthotopic tumors developed in all mice and were analyzed at 18 days post-tumor cell injection (Figure 2C-2D). Six days after tumor cell injection, mice were randomized for treatment with vemurafenib (early intervention trial). Vemurafenib treatment (tumor size mean=0.22 mm^3^, SD=0.2, p=0.01) by oral gavage for 12 days resulted in a significant reduction in tumor growth (>90%), compared with control mice treated with vehicle (tumor size mean=4 mm^3^, SD=1, p=0.01). Also, substantially decreased GFP signal suggested that this reduction was due to suppression of *BRAF^V600E^*-positive tumor cell viability and tumor growth (Figure 2C). Importantly, we found that vemurafenib therapy substantially down-regulated nuclear protein levels of cyclin B2, from about 30% of tumor cells in the vehicle-treated tumors to about 2% in vemurafenib-treated tumors (Figure 2F, Supplementary information). Although vemurafenib treatment did not significantly affect protein levels of cleaved caspase-3, marker of apoptosis (Figure 2G), we found a significant (p=0.007) increase of about 2.5-fold human lysosomal endogenous beta-galactosidase (β-gal, senescence marker) expression in the orthotopic tumor cells in the vemurafenib-treated mice (Suppl. Figure 3B) vs. vehicle-treated mice (Suppl. Figure 3A). Because *BRAF^WT/V600E^*-positive tumors tend to be more adhesive through ECM in the tumor microenvironment, we performed trichrome staining which showed a substantially abundant amount of the ECM collagen deposition surrounding the *BRAF^WT/V600E^*-KTC1 orthotopic tumor, and also intratumoral deposition of collagen; more importantly, vemurafenib anti-BRAF^V600E^ therapy substantially decreased collagen deposition in the orthtotopic tumors, reducing ECM density (Figure 2H). Furthermore, vemurafenib therapy significantly (p=0.007) reduced 2-fold vascular density (Figure 2E) and tumor microenvironment-associated PDGFRB protein levels in the *BRAF^WT/V600E^*-KTC1 tumor vs. vehicle-treated mice (Figure 2I, and Supplementary information).

### BRAF^WT/V600E^-PTC cells recruit microvascular endothelial cells and pericytes by regulating pro-angiogenic/metastatic paracrine signaling

**Figure 3:**
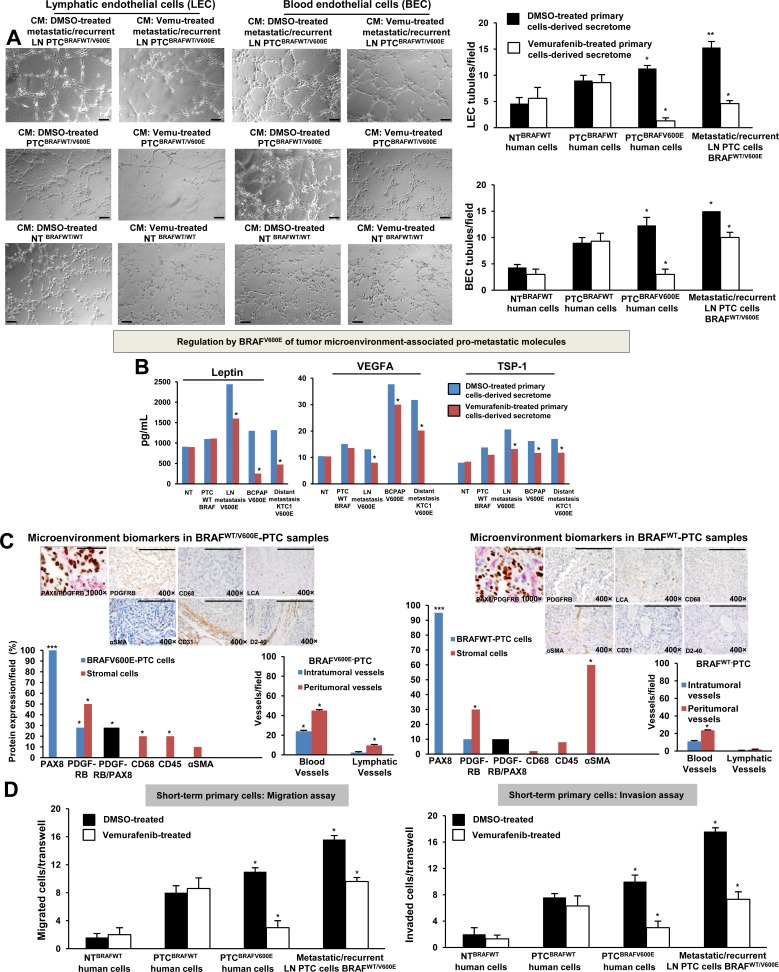
BRAF^V600E^-positive papillary thyroid carcinoma (PTC) cells promote *in vitro* angiogenesis (tubule-like structures formation) using patient-derived preclinical models **A**. *In vitro* evaluation of microvascular endothelial cell tube formation. Secretome from representative short-term primary human heterozygous *BRAF^WT/V600E^*-positive PTC triggered human lymphatic and blood microvascular endothelial cell (LEC and BEC, respectively) tube formation *in vitro*. LEC and BEC were suspended in secretome (conditioning medium (CM)) derived from short-term primary NT or PTC cells with *BRAF^WT/V600E^* or *BRAF^WT^*treated with vehicle (DMSO) or vemurafenib (10 μM) for 24 hours and seeded on growth factor–reduced Matrigel. The secretome was utilized to induce tube formation. LEC and BEC tubes were photographed after 5-6 hours. Histograms show numbers of LEC and BEC tubes triggered by non-metastatic *BRAF^WT/V600E^* PTC-derived secretome, metastatic/recurrent *BRAF^WT/V600E^* PTC-derived secretome, or in the presence of secretome derived from primary NT cells or PTC cells with *BRAF^WT^*. Magnification: 10×. All scale bars are=200 μ. These data represent the average ± standard deviation (error bars) of 2 independent experiments replicate measurements (*p < 0.05, **p < 0.01, ***p < 0.001, Mann-Whitney test). **B**. Measurements of Leptin, VEGFA and TSP-1 secreted protein levels in the vehicle-treated or vemurafenib-treated secretome derived from representative: short-term primary NT cells, short-term primary metastatic/recurrent *BRAF^WT/V600E^*-PTC cells, spontaneously immortalized human PTC-derived BCPAP cells (homozygous *BRAF^V600E^*) and metastatic PTC-derived spontaneously immortalized human KTC1 cells (heterozygous *BRAF^V600E^*). NT or PTC cells were cultured *in vitro* in the presence of vemurafenib (10 μM) or vehicle (DMSO) for 24 hours. The secretome (growth medium enriched with PTC or NT-derived growth/angiogenic factors) was collected and protein levels (pg/mL) were determined by ELISA (enzyme-linked immunosorbent assay). Protein levels were normalized to total protein content (μg/μL). These data represent the average ± standard deviation (error bars) of 2 independent experiments replicate measurements (*p < 0.05, Mann-Whitney test). **C**. Assessment of PTC microenvironment in formalin-fixed paraffin-embedded clinical human PTC specimens harboring *BRAF^WT/V600E^* (n=4) versus *BRAF^WT^* (n=3). Immunohistochemical protein expression of: PAX8 (marker of PTC cells) showed nuclear localization; PDGFRB (platelet-derived growth factor receptor beta, pericyte lineage marker and tumor cell marker) showed cytoplasmic/plasma membrane localization; CD68 (CD68, macrophage marker), localized to plasma membrane; CD45 (also called LCA, leucocyte lineage marker) localized to plasma membrane; and αSMA (alpha Smooth Muscle Actin, markers of smooth muscle cell lineage) mainly localized to plasma membrane. Black bars indicate percent of colocalization between PDGFRB and PAX8. Immunohistochemical protein expression per field was assessed semi-quantitatively using the following scoring method: 0 (negative), 1+ (1–10% positive cells), 2+ (11–50% positive cells), and 3+ (more than 50% positive cells). Microvascular density is defined by number of vessels per microscope field showing CD31 (BEC marker) or D2-40 (Podoplanin, LEC marker) staining. Scale bars are=500 μ (1000× magnification image) and 400 μ (400× magnification images). These data represent the average ± standard deviation (error bars) of independent replicate measurements (*p<0.05, Mann-Whitney test). **D**. Migration and invasion assays with vemurafenib or DMSO (vehicle, control) treatment in representative short-term primary human PTC cell cultures with heterozygous *BRAF^WT/V600E^* or *BRAF^WT^*, and in short-term primary human NT cell cultures. Treatment with vemurafenib at 10 μM was performed in the migration and invasion assays. These data represent the average ± standard deviation (error bars) of 2 or 3 independent experiments replicate measurements (*p < 0.05, Mann-Whitney test).

We sought to test the hypothesis that BRAF^WT/V600E^ by hyper-phosphorylation of the ERK1/2 triggers PTC lympho-angiogenesis by means of recruitment of human blood and lymphatic microvascular endothelial cells (BEC and LEC, respectively) (Suppl. Figures 4A-4B) and pericyte (Suppl. Figures 4C-4D), which are fundamental cell populations in the tumor microenvironment. We developed a trans-well endothelial cell migration *in vitro* assay based on PTC- or NT-derived secretome (Suppl. Figure 4A-4B) which revealed that *BRAF^WT/V600E^*^−^PTC cells promoted pericytes (Suppl. Figure 4C, Suppl. information) and endothelial cell migration (Suppl. Figure 4E, Suppl. Figure 4H, Suppl. information), and angiogenesis (i.e. *in vitro* tubule formation) (Figure 3A, Suppl. Figure 4G, Suppl. information), suggesting activation of potential pro-metastatic paracrine signaling. Tubule formation decreased (p=0.02) 1.5-3.3 fold in the presence of secretome derived from 10 μM vemurafenib-treated metastatic/recurrent *BRAF^WT/V600E^*-PTC cells compared to vehicle-treated cells; whereas, it was strongly inhibited from about 4-fold up to 8.6-fold in the presence of vemurafenib-treated secretome derived from non-metastatic *BRAF^WT/V600E^*-PTC cells (Figure 3A, Suppl. Figure 4G). LEC tubule formation strongly decreased (9.7-fold) in the presence of 10 μM vemurafenib-treated secretome derived from *BRAF^WT/V600E^*-KTC1 cells compared to vehicle-treated secretome derived from KTC1 cells (Suppl. Figure 4G). No statistically significant changes in tubules formation were observed in LEC or BEC grown in the presence of secretome derived from either *BRAF^WT^*-PTC cells or NT cells (Figure 3A, Suppl. Figure 4F). Accordingly, we found that some pro-angiogenic and ECM remodeling genes showed moderate or high mRNA expression levels in *BRAF^V600E^*-PTC cells compared with *BRAF^WT^-*PTC or NT cells (Suppl. Table 4). Vemurafenib treatment reduced their mRNA expression levels compared with *BRAF^V600E^*-PTC vehicle-treated cells (p<0.05) (Suppl. information, Suppl. Table 4).

We also used a multiplex ELISA assay that included the most known pro-angiogenic and anti-angiogenic factors. We found that LN metastatic/recurrent *BRAF^WT/V600E^*-PTC cells secreted significantly higher protein levels of pro-angiogenic factors (e.g. Leptin, ∼2-fold) *in vitro* compared with *BRAF^WT^*-PTC cells (p=0.01) or NT cells (p=0.01) (Figure 3B). Mechanistically, we found that the rate of tubule suppression by the human Leptin blocking antibody was even more potent (4.3-fold decrease, p=0.02) when BEC were grown in the presence of 10 μM vemurafenib-treated secretome derived from primary metastatic/recurrent *BRAF^WT/V600E^*-PTC cells (Suppl. Figures 4J), suggesting that inhibition of both BRAF^V600E^ and Leptin pathways was more effective to reduce angiogenesis *in vitro*. Tumor cell migration/invasion is functionally linked to angiogenic paracrine communication between tumor cells and stromal cells; indeed, we also assessed microenvironment-associated stromal cell types (Figure 3C) which participate in the aberrant behavior of metastatic *BRAF^V600E^*-PTC cells using a co-localization approach for PDGFRB and PAX8 (thyrocyte cell marker) proteins; we found that PDGFRB protein levels were significantly higher (2.2-fold increase, p=0.02) in metastatic *BRAF^V600E^*-PTC-associated stromal cells (i.e. mesenchymal stem cell–like pericytes) compared to *BRAF^WT^*-PTC cells in clinical samples (Figure 3C and Supplementary information). Remarkably, vemurafenib therapy significantly (p=0.01) reduced cell migration and invasiveness by approximately 3.5-fold in non-metastatic *BRAF^WT/V600E^*-PTC cells *in vitro* as compared with vehicle-treated (control) cells (Figure 3D). In contrast, metastatic/recurrent *BRAF^WT/V600E^*-PTC cells (Figure 3D and Suppl. Figure 6A-6B, Supplementary information) were less responsive to vemurafenib treatment, with 1.5-fold or 2-fold decrease in migration or invasion. Treatment with vemurafenib did not affect migration or invasion of *BRAF^WT^*-PTC and NT cells.

### Alterations of *MCL1* and *P16* somatic copy number in human PTC samples and PTC cell cultures

**Figure 4:**
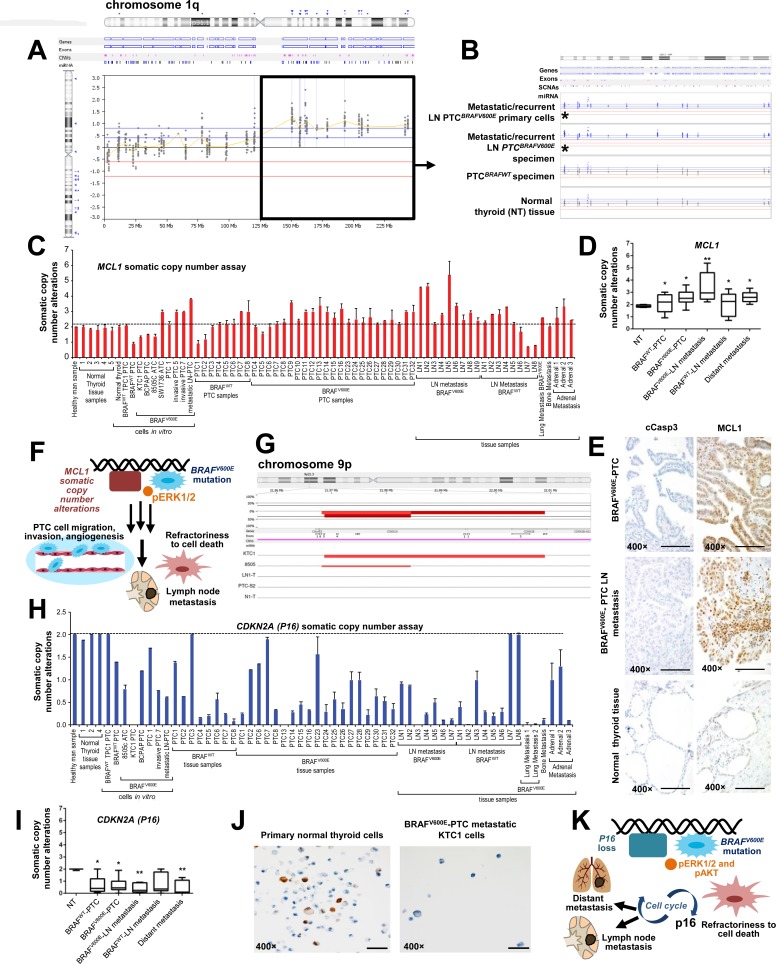
Analysis of MCL1 somatic copy number gain (chromosome 1q) and loss of P16 (chromosome 9p) in non-metastatic or metastatic papillary thyroid carcinoma (PTC) samples **A**. Graphical representation of the log2 ratio of sequence coverage in tumor versus reference for fragments sequenced using a targeted exome sequencing strategy. *BRAF^V600E^*-PTC primary cells or patient specimens harboring the BRAF^V600E^ mutation were compared with PTC or NT samples with *BRAF^WT^*. This analysis revealed 1q somatic copy number alterations (SCNAs) (i.e. copy number gain/amplifications) in metastatic/recurrent neck or mediastinal lymph nodes (LN). **B**. Detailed view of sequenced exons in 1q, including the *MCL1* gene highlighted by asterisks, in metastatic/recurrent mediastinal LN (lymph node) *BRAF^V600E^*-PTC primary cells and patient specimens harboring the *BRAF^V600E^* mutation compared with PTC or NT samples with *BRAF^WT^*. Probes that were colored or shaded blue or marked by blue triangles were called as gained by the analysis software. **C**. *MCL1* somatic copy number analysis normalized in 31 samples which included PTC and normal thyroid samples, and some established primary PTC or NT cell cultures derived from same patients' cohort, from independent patients. Histogram shows *MCL1* copy number assay results in: 1 DNA control sample from healthy man; 1 primary NT (normal thyroid) cell culture; 3 primary PTC cell cultures (2 with *BRAF^WT/V600E^* and 1 with *BRAF^WT^*), 1 mediastinal LN metastatic *BRAF^V600E^*-PTC cell culture; 5 spontaneously immortalized cell lines established from patients with *BRAF^V600E^* (i.e., KTC1 and BCPAP) or *BRAF^WT^*(TPC1) PTC, or *BRAF^V600E^*-positive anaplastic thyroid cancer (ATC) (i.e. 8505c and SW1736); 5 NT (normal thyroid) tissue samples, 31 PTC (23 *BRAF^V600E^*-PTC and 8 *BRAF^WT^*-PTC), 17 LN metastatic PTC (9 *BRAF^V600E^*-LN and 8 *BRAF^WT^*-LN) and 5 distant metastatic samples from patients with PTC (1 from lungs, 1 from bone and 3 from adrenal glands). Results were normalized against two separate reference, housekeeping (reference) genes: *GAPDH* and *RNAase-P*. These data represent the average ± standard error mean (error bars) of 2–3 independent replicate measurements. **D**. Box plot analysis using: 5 NT (normal thyroid) tissue samples, 31 PTC (23 *BRAF^V600E^*-PTC and 8 *BRAF^WT^*-PTC), 17 LN metastatic PTC (9 *BRAF^V600E^*-LN and 8 *BRAF^WT^*-LN) and 5 distant metastatic samples from patients with PTC (1 from lungs, 1 from bone and 3 from adrenal glands). These data represent the average ± standard deviation of 2–3 independent replicate measurements (*p < 0.05, **p < 0.01, ***p < 0.01, one-way ANOVA test). **E**. Immunohistochemistry shows strong and diffuse MCL1 protein expression in the cytosol and stippled nuclear staining in all neck LN metastatic PTC samples harboring *BRAF^V600E^* (n=3) (scoring, 3+) and primary PTC with *BRAF^V600E^* (n=4) (scoring, 3+), whereas NT samples (n=3) (scoring, 1+) showed focal and weak nuclear MCL1 localization. Primary *BRAF^V600E^*-PTC (scoring, 0) or LN metastatic *BRAF^V600E^*-PTC (scoring, 0) samples did not show significant change in cytoplasmic cleaved caspase 3 (cCasp3) protein levels compared with NT samples (scoring, 0). Protein expression was assessed semi-quantitatively using the following scoring method: 0 (negative), 1+ (1–10% positive cells), 2+ (11–50% positive cells), and 3+ (more than 50% positive cells). All scale bars are=400 μ. **F**. Proposed mechanisms of LN metastatic PTC spreading from primary PTC harboring the *BRAF^V600E^* mutation along with *MCL1* copy number gain/amplification. **G**. Graphical representation of next generation sequencing, targeted-exome sequencing results showing loss of *P16* (marked by red bars) in the chromosome 9p in PTC (i.e. heterozygous *BRAF^WT/V600E^*-positive human KTC1 PTC-derived cells with *P16* homozygous loss) or in ATC samples with distant metastasis (i.e. homozygous *BRAF^V600E^*-positive 8505c ATC cells) compared with metastatic/recurrent LN *BRAF^V600E^*-PTC sample (LN1-T) or derived primary cells (e.g. LN0-PTC), *BRAF^V600E^*-PTC sample (e.g. PTC-7), *BRAF^WT^*-PTC sample (e.g. PTC-S2), or NT sample with *BRAF^WT^* (e.g. N1-T) which show neutral copy number without loss of *P16*. The top red bar shows the aggregate SCNAs for all samples. **H**. *P16* somatic copy number alteration analysis normalized in: 1 DNA control sample from healthy man; 3 primary PTC cell cultures (2 with *BRAF^WT/V600E^* and 1 with *BRAF^WT^*), 1 mediastinal LN metastatic *BRAF^V600E^*-PTC cell culture; 4 spontaneously immortalized cell lines established from patients with *BRAF^V600E^* (i.e., KTC1 and BCPAP) or *BRAF^WT^*(TPC1) PTC, or *BRAF^V600E^*-positive anaplastic thyroid cancer (ATC) (i.e. 8505c); 4 NT (normal thyroid) tissue samples, 27 PTC (19 *BRAF^V600E^*-PTC and 8 *BRAF^WT^*-PTC), 15 LN metastatic PTC (7 *BRAF^V600E^*-LN and 8 *BRAF^WT^*-LN), 6 distant metastatic samples from patients with PTC (2 from lungs, 1 from bone and 3 from adrenal glands). Results were normalized against two separate reference, housekeeping (reference) genes: *GAPDH* and *RNAase-P*. Histogram shows *P16* copy number assay. These data represent the average ± standard error mean (error bars) of 2–3 independent replicate measurements. **I**. Box plot analysis using: 4 NT (normal thyroid) tissue samples, 27 PTC (19 *BRAF^V600E^*-PTC and 8 *BRAF^WT^*-PTC), 15 LN metastatic PTC (7 *BRAF^V600E^*-LN and 8 *BRAF^WT^*-LN), 6 distant metastatic samples from patients with PTC (2 from lungs, 1 from bone and 3 from adrenal glands). These data represent the average ± standard deviation of 2–3 independent replicate measurements (*p < 0.05, **p < 0.01, ***p < 0.01, one-way ANOVA test). **J**. Immunocytochemistry shows loss of P16 protein expression in human KTC1 PTC-derived cells (*BRAF^WT/V600E^* spontaneously immortalized metastatic PTC cells) compared with primary NT cells which show P16 protein focal staining in the nuclei. All scale bars are=100 μ. **K**. Proposed mechanisms of metastatic spreading from primary PTC harboring the *BRAF^V600E^* mutation along with loss of *P16*.

We have used a new algorithm (see methods) for detecting somatic mutations, insertions, deletions, copy number gain (amplifications), copy number loss, and translocations using a targeted exome sequencing strategy. We found significantly (p=0.0001) that chromosome 1q (Figure 4A-4B, Suppl. Figure 7) showed SCNAs (i.e. copy number gain) of 26 genes (Suppl. Table 3) and chromosome 9p21.3 showed loss of the *P16* gene in PTC (Suppl. Table 2, Figure 4G, Suppl. Figure 7). Here, we have focused our attention and validations on *MCL1* (a BCL2 protein family member) (one of the 26 genes with copy number gains on chromosome 1), because it is a well-known key-player in cancer progression and metastasis [21], but has not been linked to metastatic *BRAF^V600E^*-PTC. To validate this finding, we used quantitative PCR (Figure 4C-4D) using 58 independent samples (including 22 patient-derived independent metastatic PTC clinical samples, Suppl. Table 2, Figure 4C), in particular: 31 PTC patients specimens (23 *BRAF^V600E^*-PTC and 8 *BRAF^WT^*-PTC), 17 LN metastatic PTC (9 *BRAF^V600E^*-LN and 8 *BRAF^WT^*-LN), 5 distant metastatic samples from patients with PTC (1 from lungs, 1 from bone and 3 from adrenal glands), and 5 NT (Figure 4C). One DNA control sample from healthy man (human male genomic DNA) was also used (Figure 4C). Additionally, we analyzed genomic DNA from 4 primary PTC cell cultures (3 PTC with *BRAF^V600E^* and 1 PTC with *BRAF^WT^*), 1 primary LN metastatic *BRAF^V600E^*-PTC cell culture, 1 primary NT cell culture and 5 spontaneously immortalized human thyroid cancer cells (i.e. TPC1, BCPAP, KTC1, 8505c and SW1736) (Figure 4C). We found significantly that 34 of 41 (82.9%) *BRAF^V600E^*-positive samples vs. 10 of 17 (58.8%) BRAF^WT^ samples (including primary PTC, LN metastatic PTC-derived specimens and primary short-term PTC cell cultures) harbored *MCL1* copy number gain (p<0.001), and also as compared to NT tissue samples or healthy man DNA control sample which did not show any *MCL1* copy number gain (Figure 4C-4D). Eleven of 12 (91.6%) of the primary *BRAF^V600E^*-PTC specimens with a clinical story of LN metastasis and 9 of 9 (100%) *BRAF^V600E^*-PTC LN metastatic samples in the neck or mediastinum substantially harbored *MCL1* copy number gain. Lesser significant prevalence of *MCL1* copy number gain/amplification was found in *BRAF^WT^*-PTC LN metastatic samples (5 of 8, 62.5%) as compared to the *BRAF^V600E^*-PTC LN metastatic samples (p<0.01). Also, 4 of 5 (80%) PTC samples with distant metastasis showed *MCL1* copy number gain (Figure 4C-4D); whereas, NT samples did not show *MCL1* copy number gain.

As further validation, high copy number of *MCL1* gene also substantially associated with higher *MCL1* mRNA expression levels (about 1.5-2.3-fold) in metastatic *BRAF^V600E^*-PTC cell cultures (98 mRNA copies/10^6^ 18S copies) vs. primary *BRAF^WT^*-PTC (64 mRNA copies/10^6^ 18S copies) or NT cells (42.7 mRNA copies/10^6^ 18S copies) *in vitro*. *MCL1* expression levels showed 104 mRNA copies/10^6^ 18S copies by vemurafenib treatment in *BRAF^V600E^*-PTC cells and 98 mRNA copies/10^6^ 18S copies in the vehicle-treated cells. There was no substantial effect of vemurafenib on *MCL1* mRNA expression in *BRAF^WT^*-PTC or NT cells. Additionally, immunohistochemistry (IHC) analysis showed substantially strong and diffuse MCL1 protein immunostaining in all neck LN metastatic *BRAF^V600E^*-PTC samples vs. primary *BRAF^V600E^*-PTC samples, *BRAF^WT^*-PTC with or NT samples (Figure 4E), further suggesting that *MCL1* SCNAs could be important for metastasis in *BRAF^V600E^*-PTC (Figure 4F).

Furthermore, we validated *P16* SCNAs (Figure 4G, 4H and 4I) using: (i) 1 control DNA sample from healthy man; (ii) 27 PTC (19 *BRAF^V600E^*-PTC and 8 *BRAF^WT^*-PTC), 15 LN metastatic PTC (7 *BRAF^V600E^*-LN and 8 *BRAF^WT^*-LN), 6 distant metastatic samples from patients with PTC (2 from lungs, 1 from bone and 3 from adrenal glands), and 4 NT (Figure 4I) tissue samples; and (iii) 2 primary PTC cell cultures (2 PTC with *BRAF^V600E^* and 1 PTC with *BRAF^WT^*), 1 primary LN metastatic *BRAF^V600E^*-PTC cell culture, and 4 spontaneously immortalized human thyroid carcinoma cells (i.e. TPC1, BCPAP, KTC1, and 8505c). These samples included 21 patient-derived independent metastatic PTC (Suppl. Table 2, Figure 4H). Our validations significantly showed that 33 of 34 (97%) *BRAF^V600E^*-positive samples vs. 14 of 18 (77.7%) BRAF^WT^ samples (including primary PTC, LN metastatic PTC-derived specimens and primary short-term PTC cell cultures) loss of the *P16* gene (p<0.001), and also as compared to NT tissue samples or healthy man DNA control sample that did not show any loss of *P16* (Figure 4G-4I, Suppl. Figure 7). Eight of 9 (88.8%) of the primary *BRAF^V600E^*-PTC specimens with a clinical story of LN metastasis, 7 of 7 (100%) *BRAF^V600E^*-PTC LN metastatic samples in the neck or mediastinum, and 7 of 7 (100%) of the *BRAF^WT^*-PTC LN metastatic samples substantially showed loss of *P16*. Six of 6 (100%) PTC samples with distant metastasis showed *P16* SCNAs compared to NT samples (Figure 4H-4I). In particular, we found *P16* heterozygous loss occurred in 13 of 60 (21.6%), and homozygous loss in 39 of 60 (65%) samples analyzed, specifically in: 13 of 19 (68.4%) *BRAF^V600E^*-PTC, 6 of 8 (75%) *BRAF^WT^*-PTC, 6 of 7 (85.7%) *BRAF^V600E^*-PTC with neck LN metastasis, 5 of 8 (62.5%) *BRAF^WT^*-PTC samples with neck LN metastasis and 4 of 6 (66.6%) samples with distant metastasis (Suppl. Table 2). Furthermore, IHC analysis showed a substantial loss of P16 protein in the metastatic *BRAF^V600E^*-PTC derived KTC1 cells (Figure 4J) vs. primary human NT cells, suggesting that this SCNA may be important for metastatic potential of *BRAF^V600E^*-PTC (Figure 4K).

### Combination of vemurafenib therapy with the pan-BCL2 inhibitor (obatoclax) increases death of angio-invasive BRAF^V600E^-PTC cells harboring *MCL1* copy number alterations and decreases intrinsic resistance to vemurafenib

**Figure 5:**
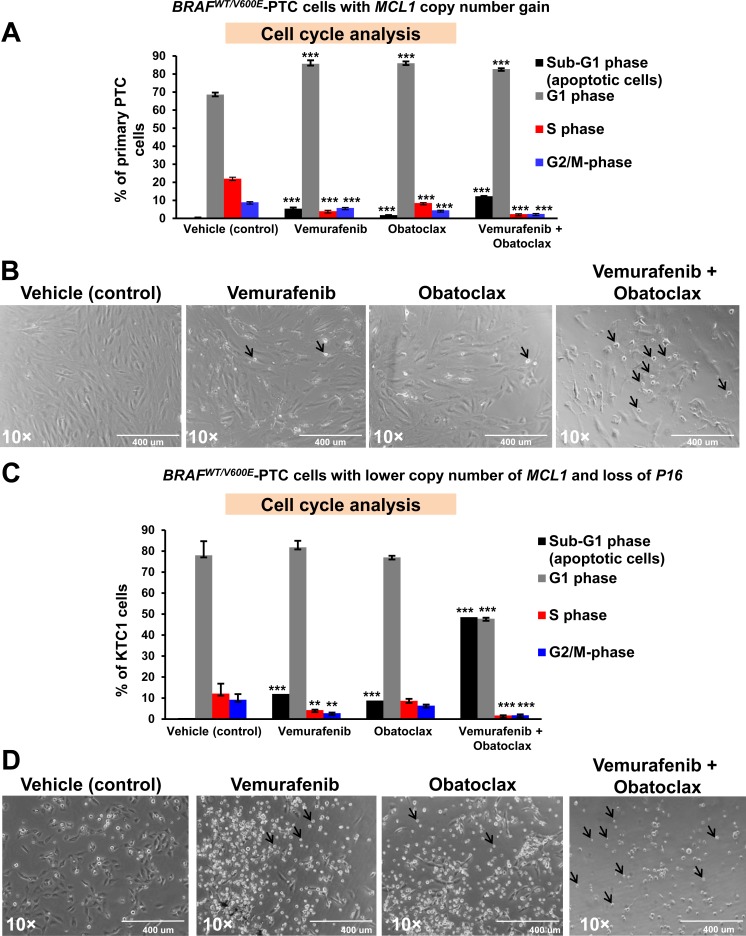
Anti-BRAF^V600E^ (vemurafenib) and anti-BCL2/MCL1 (obatoclax) combined therapy increases cell death in patient-derived angioinvasive papillary thyroid carcinoma (PTC) cells harboring BRAF^V600E^ and with somatic copy number alterations (SCNAs) of MCL1 or P16 **A**. Flow cytometry analysis of non-synchronized cells normalized to the number of events in each condition shows that 10 μM vemurafenib or 100 nM obatoclax at 48 hours post-treatment partially increased sub-G1 (apoptosis) in representative patient-derived angioinvasive PTC cells (i.e. PTC7) harboring the *BRAF^WT/V600E^* mutation and with *MCL1* copy number gain/amplifications compared to vehicle-treated (DMSO, control) cells. Five μM vemurafenib and 100 nM obatoclax combined treatment significantly increased the percent of PTC cells in sub-G1 compared to vehicle-treated (DMSO, control) cells. These data represent the average ± standard deviation (error bars) of 2 independent experiments replicate measurements (*p < 0.05, **p < 0.01). **B**. Arrows highlight change of cell shape (rounded up and detached) in angioinvasive *BRAF^WT/V600E^*-PTC cells (i.e. PTC7) with *MCL1* copy number gain/amplifications treated for about 48 hours with vemurafenib (10 μM), obatoclax (100 nM), or vemurafenib (5 μM) + obatoclax (100 nM) compared to vehicle-treated PTC cells (DMSO, control). All scale bars are=400 μ. **C**. Flow cytometry analysis of non-synchronized cells normalized to the number of events in each condition at 48 hours post-treatment shows that 10 μM vemurafenib or 100 nM obatoclax significantly increased sub-G1 in metastatic PTC patient-derived KTC1 cells harboring the *BRAF^WT/V600E^* mutation and with a lower copy number of *MCL1* and with *P16* homozygous loss compared to vehicle-treated (DMSO, control) cells. Five μM vemurafenib and 100 nM obatoclax combined treatment strongly increased the percent of KTC1 cells in sub-G1 compared to vehicle-treated (DMSO, control) cells. These data represent the average ± standard deviation (error bars) of 2 independent experiments replicate measurements (*p < 0.05, **p < 0.01, ***p < 0.01). **D**. Arrows highlight change of cell shape (rounded up and detached) in metastatic *BRAF^WT/V600E^*-KTC1 cells in the presence of vemurafenib (10 μM), obatoclax (100 nM), or vemurafenib (5 μM) + obatoclax (100 nM) compared to vehicle-treated PTC cells (DMSO, control). All scale bars are=400 μ.

Although the lack of response to vemurafenib has been frequently observed in different human cancer cells lines (e.g. melanoma cells) harboring the *BRAF^V600E^* mutation, it is still unknown in human metastatic PTC cells. To address this unmet clinical need, we used patient-derived *BRAF^V600E^*-positive angioinvasive PTC cells. Importantly, we found that angioinvasive *BRAF^WT/V600E^*-PTC cells (i.e. PTC7 cells) with *MCL1* copy number gain (Figure 4C) were more resistant to vemurafenib-induced inhibition of cell viability as compared to non-metastatic *BRAF^WT/V600E^*-PTC cells with *MCL1* neutral copy (Figure 1E-1G). Mechanistically, based on the vemurafenib IC50 calculations (Figure 1G) we found that reduced doses of 2.5 μM vemurafenib or even more effectively 5 μM vemurafenib combined with 100 nM obatoclax significantly ameliorated therapeutic response of angio-invasive *BRAF^V600E^*-PTC cells compared to single agent therapy within 48 hours (Figure 5A). This combined treatment resulted in a significant increased cell death (sub-G1 cell accumulation, indicator of apoptosis) detected by flow cytometry analysis by using single agent vemurafenib (13.5-fold) or obatoclax (4.5-fold) treatment compared to vehicle (Figure 5A). More importantly, vemurafenib plus obatoclax combined treatments significantly increased number of *BRAF^V600E^*-PTC cells in sub-G1 phase (p<0.05) as compared to single agents treatments (2.2-fold to vemurafenib and 6.8-fold to obatoclax) or to vehicle treatment (30.7-fold) (Figure 5A). Importantly, this combinatorial targeted treatment was also effective to alter *BRAF^V600E^*-PTC cell shape (cells became rounded up and detached) as compared to vehicle-treated cells (Figure 5B).

Intriguingly, metastatic *BRAF^V600E^*-PTC derived KTC1 cells (Figure 5C) with a lower copy number of *MCL1* (+/−) (Figure 4C) were more sensitive to single agents treatments or more effectively to vemurafenib plus obatoclax combined treatments compared to *BRAF^V600E^*-PTC cells with a higher copy number of *MCL1* (Figure 5C); as result, these cells significantly increased in the sub-G1 phase (apoptosis) (Figure 5C) (p<0.05). In particular, they accumulated in sub-G1 phase 24-fold by vemurafenib (10 μM), 17.6-fold by obatoclax (100 nM), and 97.1-fold by vemurafenib plus obatoclax treatments compared to vehicle (Figure 5C). Furthermore, *BRAF^V600E^*-PTC KTC1 cells with *MCL1* (+/−) (Figure 5D) showed more substantial alterations in cell shape as compared to metastatic *BRAF^V600E^*-PTC cells with a higher copy number of *MCL1* (Figure 5B).

## DISCUSSION

The pre-clinical trial model for cancer treatment may very well facilitate our ability to combat human cancer [1, 7, 22-27]. Here, we report the first patient-derived pre-clinical model of PTC with *BRAF^V600E^* or *BRAF^WT^*, or NT using anti-BRAF^V600E^ therapy. Although primary NT or PTC cell survival in culture is limited, the benefit is that primary cells show low likelihood of adopting cellular and molecular changes *in vitro*.

Our results raise the concern that primary/intrinsic resistance to *BRAF^V600E^* inhibitors (i.e. vemurafenib) could occur at or near the acquisition of metastatic capability by *BRAF^V600E^*-PTC. Critically, our experimental models identify that genomic co-occurrence of *BRAF^V600E^* mutation with either *MCL1* copy number gain (chromosome 1q) or *P16* (chromosome 9p) loss is functionally associated with metastasis in patients with PTC and patients should be closely monitored for disease recurrence. This paired genomic abnormality could confer primary/intrinsic resistance to vemurafenib therapy, possibly through activation of BRAF^V600E^ parallel signaling pathways regulated by either MCL1 or P16 in metastatic PTC cells. Furthermore, abundance of *MCL1* (pro-survival factor) or loss of *P16* (tumor suppressor) protein and mRNA levels functionally correlated with their genomic abnormalities. MCL1 is an anti-apoptotic member of the BCL2 family and is known to be focally amplified in about 10.9% of cancers across multiple tissue types (e.g. breast cancer, lung cancer, etc.) and to increase tumor cell survival [21]. MCL1 is also amplified in melanoma cells [28]. Our orthotopic mouse model of tumor harboring the heterozygous *BRAF^V600E^* mutation along with *MCL1* and *P16* copy number alterations showed focal PTC-like nuclear features (i.e. clearing), suggesting that this mouse model may be a helpful translational tool for *in vivo* pre-clinical testing of anti-BRAF^V600E^ therapy, although these tumor cells (i.e. KTC1 cells) showed *in vivo* mixed spindled and epithelioid features which are not characteristic of PTC. Vemurafenib therapy did not elicit expression of pro-apoptotic markers in the orthotopic tumor cells; interestingly, tumor cell senescence increased with concomitant tumor size reduction via suppressed pro-survival molecules. Moreover, our analysis of tumor microenvironment both in our orthotopic tumors and in PTC clinical samples provides evidence that metastatic *BRAF^V600E^*-PTC with *MCL1* and *P16* copy number alterations are enriched with PDGFRB-positive stromal cells (i.e. pericyte). Additionally, BRAF^V600E^-positive PTC cell might synergize with pericytes and sustain the viability via expression and secretion of factors crucial for angioinvasion and angiogenesis. Furthermore, our *in vitro* data indicate that BRAF^V600E^ might regulate PTC cell migration/invasion and viability, and its inhibition by vemurafenib may induce cell cycle arrest and apoptosis in non-metastatic PTC cells. In contrast, metastatic *BRAF^V600E^*-PTC cells with *MCL1* copy number gain are substantially less sensitive and responsive to vemurafenib treatment as a single agent. Consistent with our genetic data, we critically show that combined therapy with vemurafenib and pan-BCL2/MCL1 inhibitor decreased viability of angioinvasive *BRAF^V600E^*-PTC cells *in vitro*. This effect could be determined by the inhibition of BCL2 and MCL1 (1q) which are known to inhibit apoptosis and maintain cancer cell survival. Co-treatment with BRAF inhibitors and with inhibitors of BCL2 might overcome intrinsic resistance to BRAF inhibitors (also seen in melanoma cells *in vitro* and *in vivo*) [28]. BCL2 inhibitors synergize with MEK inhibitors and induce *in vitro* death of murine poorly differentiated thyroid carcinoma cells [29]. Moreover, in lung cancer, reduced *MCL1* expression sensitized epidermal growth factor receptor mutant non-small cell lung cancers to MEK inhibitors [30]. BCL2 family inhibitors, in combination with a TORC1/2 inhibitor, lead to apoptosis in *KRAS*- and *BRAF*-mutants but not wild-type (WT) colorectal cancer cells. This is accompanied by suppression of MCL1 expression in mutant, but not WT, colorectal cancers [31]. However, BCL2 inhibitors may be toxic, and therefore, ascertaining the correct dosage is essential.

Importantly, the recent PTC TCGA data show chromosome 1q amplification in aggressive BRAF^V600E^-positive PTC [5], suggesting key role for this genomic region in PTC behavior. PTC heterogeneity might affect the sensitivity for detection of copy number alterations (*MCL1*, *P16*, etc.) in some cases, with possible epigenetic alterations of MCL1 and P16 expression levels; therefore, we cannot exclude the possibility that the percentage of copy number alterations for these genes might have some variability. However, our results were substantially validated by our integrated *in vitro* functional assays and *in vivo* approaches.

Molecular screening for *BRAF* mutations, as well as *MCL1* and *P16* copy number alterations might help identify subsets of patients with PTC which have high risk for recurrence and metastasis. They may benefit from combined targeted therapies directed against these alterations. This approach might provide a novel advancement in the therapeutic strategy of aggressive and refractory *BRAF^V600E^*-PTC for cases in which surgery is limited by tumor location and extent of disease, or in medically poor surgical candidates. Short-term PTC and NT cell cultures appear to provide a novel predictive model for evaluating therapeutic responses in patients with PTC. Collectively, results from our PTC pre-clinical model provide data that have value beyond predicting the design and outcome of concurrent metastatic *BRAF^V600E^*-PTC clinical trials which are using BRAF^V600E^ inhibitors (e.g. vemurafenib therapy) and also identify potentially biomarkers (e.g. *MCL1* copy number gain, loss of *P16*) for aggressive PTC.

## MATERIALS AND METHODS

### Histology and immunohistochemistry

Histopathology evaluation of 7 classical type PTC (4 PTC with *BRAF^V600E^* and 3 PTC with *BRAF^WT^*), 3 neck LN metastatic PTC samples harboring *BRAF^V600E^* (n=3) and 3 NT tissues was performed by pathologists (P.S. and V.N.) on hematoxylin and eosin (HE)-stained formalin-fixed paraffin-embedded (FFPE) tissues. For all patients we used discarded and unidentified tissues (Beth Israel Deaconess Medical Center (BIDMC) IRB-approved exemption 4 protocol, Boston). All tissue specimens were fixed with 10% buffered formalin phosphate and embedded in paraffin blocks. These were visualized with an Olympus BX41 microscope and the Olympus Q COLOR 5 photo camera (Olympus Corp., Lake Success, NY, USA). Four μm sections of PTC or BRAF^V600E^-positive human KTC1 tumor-derived orthotopic mouse tissues (serial sections) were used for IHC. After baking overnight at +37°C, deparaffinization with xylene/ethanol and rehydration was performed. IHC analysis utilized primary antibodies (Supplementary Table 5). The sections, treated by pressure cooker for antigen retrieval (Biocare Medical, Concord, CA), were incubated at 123°C in citrate buffer (Dako Target Retrieval Solution, S1699; DAKO Corp.), cooled and washed with PBS. Antigen retrieval was performed for 60 min at room temperature. The primary antibody was detected using a biotin-free secondary antibody (K4011) (Dako Envision system) and incubated for 30 min. All incubations were carried out in a humid chamber at room temperature. Slides were rinsed with PBS between incubations. Sections were developed using 3,3-diaminobenzidine (Sigma Chemical Co.) as a substrate and were counterstained with Mayer's hematoxylin [1] [32] [33]. The IHC marker expression was assessed semiquantitatively using the following scoring method: 0 (negative), 1+ (1–10% positive cells), 2+ (11–50% positive cells), and 3+ (more than 50% positive cells). Microvascular density is defined by number of vessels per microscope field showing CD31 (marker of blood endothelial cells) or D2-40 (Podoplanin) (marker of lymphatic endothelial cells) staining. Trichrome staining was performed according to manufacturer instructions (Ventana, USA) in order to assess collagen deposition in the ECM of PTC.

### Genotyping oncomap analysis and mass extend sequenom

Genomic DNA was extracted using our previous protocol [21] from overall 13 samples from independent patients, which include: (i) 2 BRAF^V600E^-PTC, 1 BRAF^WT^-PTC, 1 mediastinal LN metastatic PTC and 1 NT available FFPE discarded/unidentified samples from 4 independent patients (Beth Israel Deaconess Medical Center (BIDMC) IRB-approved exemption 4 protocol, Boston); (ii) 2 primary PTC cell cultures with BRAF^V600E^, 1 mediastinal LN metastatic PTC cell culture with BRAF^V600E^, 1 primary PTC cell culture with BRAF^WT^ and 1 primary NT cell culture; and (iii) 3 spontaneously immortalized human thyroid cancer cells (8505c, BCPAP and KTC1, see cell cultures paragraph for more details). Briefly, 30 μm paraffin sections were lysed in denaturing buffer containing proteinase K (1 mg/ml) (Invitrogen, USA) during overnight incubation at +55°C. DNA was purified using equal volumes of a phenol*:*chloroform mixture (Invitrogen, USA) and eluted in distilled water. Genomic DNA was quantified using Quant-iT PicoGreen dsDNA Assay Kit (Invitrogen, USA) per manufacturer protocol. 250 ng of genomic DNA was used for mutation detection through Oncomap version 3, which interrogates about 1000 known mutations (including the BRAF^V600E^ mutation) in 112 validated oncogenes and tumor suppressors [34]. All genomic analyses were run in the Center for Cancer Genome Discovery (CCGD, Dana Farber Cancer Institute (DFCI), Harvard Medical School, Boston, MA, USA) [34]. Primers and probes were designed using Sequenom MassARRAY Assay Design 3.0 software, applying default multi-base extension parameters. Whole genome amplification (WGA) was performed using the GenomePlex Complete WGA kit (Sigma, USA) based on chemical fragmentation followed by adapter mediated PCR amplification. Samples were run on the mass spectrometry-based genotyping platform (Sequenom) and analyzed according to current standardized protocols [34]. Sample identity and the possible introduction of artifacts by WGA were evaluated using a 48 Single Nucelotide Polymorphisms (SNPs) panel comparing the pre-WGA to the post-WGA DNA. If ≥3 SNP discrepancies were identified between SNPs found in pre- and post-WGA samples, this sample was discarded. Validations were performed using homogeneous and sensitive Mass Extend (hME) sequenom, a multi-base extension chemistry performed on native unamplified genomic DNA [34]. For all patients, we used discarded/unidentified tissue specimens and consent for genotyping test.

### Sanger sequencing and SNaPshot mutational analysis

PCR products were generated and sequenced for the *BRAF^V600E^* mutation screening according to previous protocols [35, 36]. SNaPshot analysis covered a broad panel of mutations including the *BRAF^V600E^* and was performed according to Dias-Santagata et al. [37].

### Targeted exome sequencing

OncoPanel version 2 (OPv2) represents a recent targeted exome sequencing strategy (developed from the CCGD, DFCI, Harvard Medical School) to simultaneously detect mutations, insertions, deletions, translocations and SCNAs. We analyzed 13 samples from independent patients, in particular from: 2 *BRAF^V600E^*-PTC, 1 *BRAF^WT^*-PTC, 1 mediastinal lymph node (LN) PTC metastasis and 1 NT available FFPE discarded/unidentified samples from 4 independent patients (Beth Israel Deaconess Medical Center (BIDMC) IRB-approved exemption 4 protocol, Boston); as well as we analyzed genomic DNA from 2 primary PTC cell cultures with BRAF^V600E^, from 1 mediastinal LN metastatic PTC cell culture with BRAF^V600E^ from 1 primary PTC cell culture with *BRAF^WT^*and from 1 NT cell culture. Additionally, we analyzed DNA from 3 spontaneously immortalized human thyroid cancer cells (8505c, BCPAP and KTC1 see cell cultures paragraph for more details). Targeted sequencing was achieved by designing RNA baits to capture the exons of 504 genes with relevance to cancer. The bait set was augmented with specific intronic sequences to also detect translocations often involved in cancer. Sequencing libraries were prepared, as previously described in Hettmer et al. [38], starting from 100 ng of genomic DNA. Libraries were quantified by qPCR (Kapa Biosystems, Inc, Woburn, MA) and pooled in equimolar concentrations to 500 ng total and enriched for the OPv2 bait set using the Agilent SureSelect hybrid capture kit. The enriched targeted exon libraries were again quantified by qPCR (Kapa Biosystems, Inc, Woburn, MA) subsequently sequenced in one lane of a Hiseq2000 sequencer (Illumina Inc, San Diego, CA) in a 2× 100 bp pair-end mode. Sequence alignment, demultiplexing and variant calling, including SCNAs and Indels (insertions-deletions) were performed using PICARD (HTSJDK Java library HTSJDK, BROAD Institute, Cambridge, MA, USA), GATK tools, Mutect and IndeLocator as previously described in Hettmer et al. [38]. Common variants were filtered against the 6,500 exome release of the Exome Sequencing Project (ESP) database. Variants represented in either the African-American or European-American and not in COSMIC > 2× were considered to be germline. The ESP filter was only applied to tumor samples that did not have a matched normal DNA sample.

### Somatic copy number alteration analysis

Somatic copy number alterations (SCNAs) analysis was performed using Nexus7.1 (BioDiscovery Inc., USA) after calculating the sequencing coverage using PICARD. Coverages were normalized over GC-content using a loess regression and DNA derived from normal and immortalized cultured lympho-blastoid cells by the CEPH (Centre d'Etude du Polymorphisme Humain; http://www.cephb.fr/) as reference. SCNAs were called with the following next generation sequencing (NGS) settings: significant threshold = 1E-6; max contiguous probe spacing or 1000 Kbp; minimum number of probes per segment = 3. SCNA gains has a log ration >0.4 and were called high gain if >0.8. Single copy loss threshold was −0.6 and big loss was −1.2. SCNA's on chromosomes X and Y were not called.

### Translocations analysis

Translocations (rearrangements) were detected by Breakmer, an algorithm that was specifically developed for targeted sequencing by the CCGD (DFCI, Harvard Medical School). Briefly, the method identified the reads that partially aligned to the targeted regions captured and sequenced. The unaligned portion of these ‘soft-clipped’ reads was used to assemble contigs. The contigs were aligned to the reference genome, and structural variants were called based on the alignment. For translocation events, discordantly mapped paired-end reads were identified which flank putative breakpoints.

### Validations of MCL1 and P16 SCNAs

Genomic DNA was extracted using our previous protocol [21] from either 58 (for *MCL1* copy number assays, see below) or 51 (for *P16* copy number assays, see below) FFPE archived clinical samples; in details, for *MCL1* copy number assays we used: 31 PTC (23 *BRAF^V600E^*-PTC and 8 *BRAF^WT^*-PTC), 17 LN metastatic PTC (9 *BRAF^V600E^*-LN and 8 *BRAF^WT^*-LN), 5 distant metastatic samples from patients with PTC (1 from lungs, 1 from bone and 3 from adrenal glands), and 5 NT; and 1 control sample from healthy man (human male genomic DNA from Promega, USA). For *P16* copy number assays we used: 27 PTC (19 *BRAF^V600E^*-PTC and 8 *BRAF^WT^*-PTC), 15 LN metastatic PTC (7 *BRAF^V600E^*-LN and 8 *BRAF^WT^*-LN), 6 distant metastatic samples from patients with PTC (2 from lungs, 1 from bone and 3 from adrenal glands), and 4 NT; and 1 control sample from healthy man. Moreover, our SCNAs analyses also included genomic DNA from 4 primary PTC cell cultures (3 PTC with *BRAF^V600E^* and 1 PTC with *BRAF^WT^*), 1 primary LN metastatic *BRAF^V600E^*-PTC cell culture, 1 primary NT cell culture and 5 spontaneously immortalized human thyroid cancer cells (i.e. TPC1, BCPAP, KTC1, 8505c and SW1736). MCL1 and P16 SCNAs analysis was respectively performed in 22 or 21 patient-derived independent metastatic PTC clinical samples (Suppl. Table 2). DNA quality (260/280 ratio) and concentration (ng/μL) were assessed by NanoDrop (ND-1000 Spectrophotometer, Thermo Fischer Scientific Inc, MA, USA); or assessed by Qubit 2.0 fluorometer (Invitrogen-Life Tech., Inc., USA) using the double-stranded DNA broad range assay kit (Invitrogen-Life Tech., Inc., USA). Samples were then diluted in order to generate uniform DNA concentrations (5 ng/μL or 20 ng/μL per replicate/reaction). For the duplex real-time PCR reaction, we used a TaqMan based copy number assay specific for: (i) *MCL1* (myeloid cell leukemia sequence 1, gene aliases: *BCL2L3*, *EAT*, *MCL1-ES*, *MCL1L*, *MCL1S*, *Mcl-1*, *TM*, *bcl2-L-3*, *mcl1/EAT*) (NCBI location: Chr.1:150547027-150552214; assay gene location: exon 1; cytoband: 1q21.3; assay reference genome Location: Chr.1:150552073 on NCBI build 37) (Life Technologies, USA, assay ID: Hs01326481_cn, cat#4400291) in 58 samples and (ii) *P16* (cyclin-dependent kinase inhibitor 2A or *CDKN2A*, gene aliases: *ARF, CDK4I, CDKN2, CMM2, INK4, INK4A, MLM, MTS-1, MTS1, P14, P14ARF, P16, P16-INK4A, P16INK4, P16INK4A, P19, P19ARF, TP16*) (NCBI location: Chr.9:21967751-21994490; assay gene location: exon 2; cytoband: 9p21.3; assay reference genome Location: Chr.9:21974968 on NCBI build 37) (Life Technologies, USA, assay ID: Hs00237642_cn, cat#4400291) in 51 samples for which there was sufficient DNA available. We also used the copy number reference assay human *RNAase-P* (Life Technologies, USA, cat# 4403326). Briefly, each 5 μL reaction mixture was run in a 384-well plate with 4 replicates per sample each containing 2.5 μL Universal Master Mix II, no UNG, (catalog #4440040, Life Technologies, USA), 0.25 μL copy number reference assay *RNAase-P*, 0.25 μL copy number assay for *MCL-1* or *CDKN2A*, and 2 μL DNA sample at 2.5 ng/μL. *GAPDH* (glyceraldehyde-3-phosphate dehydrogenase) was utilized as an additional reference gene. Each 5 μL reaction mixture contained 2.5 μL Universal Master Mix II, no UNG, 0.1 μL of 10 μM forward primer (5′-CCTGACCTGCCGTCTAGAAAA-3′), 0.1 μL of 10 μM reverse primer (5′-CTCCGACGCCTGCTTCAC-3′) according to Li et al. [39], 0.1 μL of 10 μM *GAPDH* probe (5′-FAM-CTGCCAAATATGATGACATCAAGA-BHQ-3′) (Integrated DNA Technologies, USA), 0.2 μL DNAse/RNAse free water and 2 μL DNA sample at 2.5 ng/μL. The target and reference copy number assays were run in a duplex on Bio-Rad Real-Time PCR System (CFX Connect™ Real-Time PCR Detection System, BioRad, USA) or using the 7900HT Real-Time PCR system (Life Technologies, USA) with the program as 10 minutes polymerase activation at 95°C followed by 40 cycles of 95°C for 15 seconds and 60°C for 1 minute. The Real-Time PCR data were imported to CopyCaller^TM^ Software v2.0 (Life Technologies, USA) for comparative Ct (ΔΔCt) relative quantification analysis using a control sample (Human Male Genomic DNA from Promega, USA) with assumed 2 copies (diploid DNA) of target gene in the following equation: copy number = *cn_c_*2^−ΔΔCt^. Cn_c_ is the copy number of the target sequence (i.e. *MCL1*) in the calibrator sample (human male genomic DNA); ΔCt is the mean difference between Ct of target assay (*MCL1*) and reference assay (*GAPDH* or *RNase-P*); and ΔΔCt is the difference between ΔCt for the tested sample and the calibrator sample. We used the following threshold similarly to previous studies [40] [41] for the copy number gain/amplification assay and classified as low-level, moderate-level and high-level amplification, specifically as follows: normal copy number (1.85-2.15 fold), heterozygous deletion (0.90-1.84 fold), homozygous deletions (below 0.9 fold), gain (2.16-2.39 fold), low-level amplification (2.4-3.9 fold), moderate-level amplification (4-9.8 fold) and high-level amplification (greater than 9.8 fold) according to the distributions in reference to normal samples with diploid DNA. Assays were performed at least in duplicate.

### Cell cultures

Primary human PTC or NT cells were derived according to procedures used in previous studies [42] [43] from 0.1 to 1 gram excess fresh PTC tissue, or normal fresh thyroid tissue from patients undergoing thyroidectomy (IRB approved for AF). Briefly, we have adopted some changes in the protocol [42] [43] using 1:1 DMEM:Ham's F12 (Corning, USA) with 10% fetal bovine serum (FBS). We cut tissue into 1 mm^3^ in a sterile petri dish with cold Hanks Balanced Salt Solution (calcium and magnesium free) (HBSS) (Fischer Scientific, MA, USA). We transferred minced tissue to a 50 ml tube with HBSS. We decanted supernatant red cells. Tissue was digested in a solution with clostridium collagenase (100 U/ml) and Dispase (1 mg/ml) (Stem Cell Technologies, USA) in HBSS or in 1:1 DMEM:Ham's F12 growth medium with no serum (Corning, USA) for 1.5 hours at 37C in a humidified tissue culture incubator with 5% C02. We filtered the remaining completely digested tissue and pooled supernatants through a 70 micron cell strainer (BD Falcon, USA). We rinsed the strained material with 5 ml of HBSS to maximize yield. This straining step is important to help remove fibroblasts and blood vessels. We spun down the filtered cells at low speed and resuspended them in 1:1 DMEM:Ham's F12 with 10% FBS plus ampicillin/streptomycin antibiotic and antimycotic solution 100× (Corning, USA) for seeding at about 1×10^5^ cells per mL on plastic petri dishes. PTC cells digest faster than NT cells, and adhere relatively poorly to glass or plastic. Cell viability was generally high and cytokeratin staining confirmed >99% of adherent cells are epithelial using this method. PTC genomic status was determined by genomic sequencing (IRB approved for CN). Thyroid and epithelial cell IHC markers were validated in previous studies [23]. Human microvascular endothelial cells (human blood endothelial cells (BEC) and lymphatic endothelial cells (LEC)) were kindly provided from Dr. Harold F. Dvorak (BIDMC, Harvard Medical School, Boston, USA). Pericytes were obtained from Promo Cell (Heidelberg, Germany). Primary PTC or NT cells were grown in 1:1 DMEM:HAM's F12 (Corning, USA) supplemented with 10% FBS and ampicillin/streptomycin antibiotic and antimycotic solution 100× (Corning, USA). BEC and LEC were grown in EGM2 bullet kit (Lonza, USA) growth medium with 10% FBS. We used the following spontaneously immortalized human thyroid cancer cells: TPC-1 (PTC, harboring *RET/PTC-1* and *BRAF^WT^*) was provided by Dr. F. Frasca (University of Catania, Italy); the BCPAP cells which harbor the homozygous *BRAF^V600E^* mutation were established from the primary tumor of a 76-year-old woman with poorly differentiated PTC [44] and provided by Dr. G. Damante (University of Udine, Italy); the KTC1 cells which harbor the heterozygous *BRAF^WT/V600E^* mutation were established from the metastatic pleural effusion from a recurrent and radioiodine (RAI) refractory PTC in a 60-year-old male patient [45] by Dr. J. Kurebayashi (Department of Breast and Thyroid Surgery Kawasaki Medical School Kurashiki, Japan) and provided by Dr. Rebecca E. Schweppe (University of Colorado, USA); and the 8505c (ATC) cells harboring the hemizygous/homozygous *BRAF^V600E^* mutation were purchased from DSMZ (German collection of microorganisms and cell culture, Braunschweig, Germany) [24, 46]. These human thyroid cancer cells were validated by genomic/genotyping analyses (e.g. *BRAF^V600E^* mutation analysis, see above) and were grown in high glucose DMEM (Corning, USA) medium supplemented with 10% FBS and plus ampicillin/streptomycin antibiotic and antimycotic solution 100× (Corning, USA). All *in vitro* assays were performed by growing PTC or NT cells in growth medium supplemented with 0.2% FBS. The human embryonic kidney (HEK) 293T cell line was kindly provided by Dr. Laura E. Benjamin (ImClone Systems, NYC, USA) and grown in DMEM supplemented with 10% FBS and plus ampicillin/streptomycin antibiotic and antimycotic solution 100× (Corning, USA).

### Three-dimensional (3D) cell cultures assays

In 3D cell cultures, growth of PTC cells resulted in formation of cell aggregates (e.g. spheroids) that recapitulate some aspects of the *in vivo* ECM. Using this assay in human breast carcinoma models, it was shown that oncogenes during steps of tumor progression could elicit aggressive cell phenotype [47]. 3D cell cultures were prepared as follows: 4×10^3^ or 20×10^3^ cells were suspended in high glucose DMEM growth media supplemented with 10% FBS and seeded on growth factor-reduced Matrigel (BD Biosciences, USA) in 24-well petri dishes. Phase-contrast images (10× and 20× magnification) were captured using a microscope (Nikon TE300, USA) equipped with a camera (Leica DFC 350 FX, USA).

### Vemurafenib preparation

Vemurafenib (PLX4032, RG7204) (Roche, NYC, USA) was dissolved in absolute dimethyl sulfoxide (DMSO, vehicle) (Sigma, USA) to achieve a stock concentration of 10 mM for *in vitro* assays. Ten mM was diluted to 2 mM in absolute DMSO; then, vemurafenib intermediate doses were diluted in 0.2% FBS high glucose DMEM in order to achieve final concentrations of 0.01 μM, 0.1 μM, 1 μM, 2.5 μM, 5 μM, and 10 μM vemurafenib at 2% DMSO for optimal vemurafenib solubility. Vehicle was 2% DMSO diluted in 0.2% FBS high glucose DMEM. For *in vivo* studies, a drug suspension was prepared from micro-precipitated bulk powder (MBP) by suspending the drug to a concentration of 25 mg active pharmaceutical ingredient (API)/mL in a 2% solution of hydroxypropylcellulose (vehicle) (Sigma, USA), according to manufacturer instructions. Freshly prepared drug suspensions were stored at 4°C and used within 24-48 hours. Mice were dosed twice daily (8 hours apart) with vehicle alone (control) or with vemurafenib suspensions at 100 mg/kg using an 18G oral gavage needle.

### Obatoclax mesylate

Obatoclax [29] also named GX15-070 (Selleckchem, USA) is an inhibitor of the BCL2 proteins family (which it does also include MCL1), was dissolved in absolute DMSO (Sigma, USA) according to the manufactures instructions and then used at 2 nM, 10 nM, 20 nM, 100 nM, 500 nM and 1 μM diluted in 0.2% FBS high glucose DMEM at final 2% DMSO. Combinatorial treatments of vemurafenib plus obatoclax were calculated based on the vemurafenib IC50 values using a decrease of the highest dose (i.e. 10 μM) of vemurafenib, specifically we combined 5 μM or 2.5 μM vemurafenib along with 2 nM, 10 nM, 20 nM, 100 nM, 500 nM or 1 μM obatoclax. All compounds (single agents or combined treatments) were diluted in 0.2% FBS high glucose DMEM at final 2% DMSO. Vehicle was 2% DMSO diluted in 0.2% FBS high glucose DMEM.

### Cell viability

Human PTC or NT cells (1×10^3^ cells/well) were cultured in growth medium containing 0.2% FBS (CellGro, USA) in a 96-well sterile culture plate (Thermo Fisher Scientific, USA). Cells were treated with or without various concentrations of vemurafenib for 48 hours. Cell viability was measured using the CellTiter-Glo luminescent cell viability assay kit (Promega, USA). The IC_50_ (50% maximal inhibitory concentration) for cell viability was determined using the following doses of vemurafenib: 0.01 μM, 0.1 μM, 1 μM, 5 μM and 10 μM.

### Cell transfections for lentivurs or retrovirus production

HEK 293T cells (5 ×10^5^) were grown in 60-mm plates and transfected using Fugene-6 (Roche) in OptiMEM (Invitrogen) for 48 hours according to manufacturer instructions.

### Over-expression technique

HIV-U6-GFP GL3B lentivirus which expresses GFP (green fluorescent protein) was kindly provided from Dr. Yutaka Kawakami (Division of Cellular Signaling, Institute for Advanced Medical Research, Keio University School of Medicine, Japan). BRAF^V600E^-pBABE-puro and pBABE-puro (empty vector) retroviral constructs were kindly provided by Dr. W.C. Hahn (Dana-Farber Cancer Institute, Harvard Medical School, Boston) and used for BRAF^V600E^ over-expression studies in NT cells. Either lentiviral or retroviral infections (viral transductions) were performed as follows: HEK 293T cells were seeded in 60-mm dishes and cotransfected the next day with each lentivirus or retrovirus and helper plasmids (i.e., gag-pol and VSV-G were used only for lentiviral infections and provided by Dr. W.C. Hahn). Both pBABE retroviral constructs were similarly packaged using helper plasmid pCL-ampho provided by Dr. W.C. Hahn. Media with progeny virus from transfected HEK 293T cells were collected 48 hours later and filtered with 0.45 μm filters (Millipore) and freshly used to transduce KTC1 cells for 3–6 hours in the presence of 8 μg/mL polybrene (Sigma). All cells used were transduced at a multiplicity of infection of either 50 or 100. Finally, stable transduced cells with HIV-U6-GFP GL3B were selected and sorted by flow cytometry (MoFlo/FACSAria Sorting; Beckman Coulter); and NT cells transduced with pBABE-puro retroviral constructs were treated with puromycin (1 μg/mL) (Sigma-Aldrich) for selection. BRAF^V600E^ over-expression in the cells were confirmed by real-time RT-PCR according to Nucera et al. [1]. All assays were performed in duplicate.

### ELISA

PTC or NT cells were cultured at about 80% confluence in 6-well dishes in 1:1 DMEM/Ham's F12 or high glucose DMEM supplemented with 10% FBS and then treated with 10 μM vemurafenib or vehicle for 24 hrs in 1:1 DMEM/Ham's F12 or high glucose DMEM supplemented with 0.2% FBS. After 24 hours, the growth medium enriched with PTC or NT cell-derived growth/angiogenic factors (secretome) was collected, removed from dead cell debris by short spin, diluted 1:3, and then secreted VEGFA, FGFβ, EGF, Leptin, TNFα, IL6, IGF-1, TGFβ (protein levels, pg/mL) were determined by ELISA (Enzyme-linked immunosorbent) assay (Signosis, CA, USA) according to manufacturer instructions. TSP-1 protein levels were also determined by ELISA assay (R&D Systems, MN, USA) according to manufacturer instructions. Growth medium was measured by ELISA to subtract background and normalize all samples. Protein levels were also normalized to total protein content (μg/μL).

### Cell cycle analysis

PTC-derived cells were cultured at about 60-80% confluence in 6-well dishes in 1:1 DMEM/Ham's F12 or high glucose DMEM supplemented with 10% FBS then treated with 10 μM vemurafenib, 100 nM obatoclax, combination of 5 μM vemurafenib plus 100 nM obatoclax, or vehicle for 48 hrs in 1:1 DMEM/Ham's F12 or high glucose DMEM supplemented with 0.2% FBS. After 48 hours, adherent cells were trypsinized and pelleted with the supernatant. Cell pellets were fixed in pre-chilled (−20°C) ethanol 75%, pelleted at 400 × g at room temperature, suspended in 0.5% PBS/BSA, incubated in 2M HCl 0.5% BSA for 20 min at room temperature, washed with 0.5% PBS/BSA, and centrifuged 5 minutes at 400 × g at room temperature. The cells were suspended in 0.1M sodium borate (pH 8.5) for 2 min at room temperature, washed with 0.5% PBS/BSA, and centrifuged 5 min at 400 × g at room temperature. Finally, the cells were pelleted, washed twice with 0.5% PBS/BSA, and suspended in 500 μL of this solution. Propidium iodide (Sigma) was added to a final concentration of 10 μg/mL with RNase 10 mg/mL (Sigma). Cells were incubated at room temperature for 30 minutes and then analyzed by flow cytometry on a FACS Gallios (Beckman Coulter, Miami, FL, USA).

### Apoptosis assay

PTC-derived cells were cultured at about 60-80% confluence in 6-well dishes in 1:1 DMEM/Ham's F12 or high glucose DMEM supplemented with 10% FBS then treated with 10 μM vemurafenib, 100 nM obatoclax, combination of 5 μM vemurafenib plus 100 nM obatoclax, or vehicle for 48 hrs in 1:1 DMEM/Ham's F12 or high glucose DMEM supplemented with 0.2% FBS. After 48 hours, adherent cells were trypsinized and pelleted with the supernatant. Cell pellets were fixed in pre-chilled (−20°C) ethanol 75% for propidium iodide staining and flow cytometry analysis a FACS Gallios (Beckman Coulter, Miami, FL, USA) to evaluate sub-G1 cell population (apoptosis).

### Western blotting

Cells were cultured at about 90% confluence in 6-well dishes in 1:1 DMEM/Ham's F12 or high glucose DMEM supplemented with 10% FBS and then treated with 10 μM vemurafenib or vehicle for 24 hrs in 1:1 DMEM/Ham's F12 or high glucose DMEM supplemented with 0.2% FBS. After 24 hours, adherent cells were lysed along with the pelleted supernatant. Western blotting assays were performed according to a standard procedure, and the protein lysis buffer was composed of 10 mM Hepes (pH 7.40), 150 mM NaCl, 5 mM EDTA, 1 mM EGTA, 1 mM sodium vanadate, 5 mM sodium flouride, and 1% Triton X-100; protease and phosphatase inhibitors (Pierce) were used for protein extractions [1]. The intensity of the bands was quantified with a densitometer (Quantity One 1-D analysis software, Bio-Rad, USA). The quantity of signal in the vemurafenib lane was divided by the signal of the vehicle (control) lane in the corresponding tubulin or β-actin blot lane. The assessment of the 90% maximal inhibitory concentration (IC_90_) of ERK1/2 phosphorylation was performed by densitometry comparing phospho-ERK1/2 protein expression levels upon 10 μM vemurafenib treatment vs. vehicle (control) treatment by densitometry. We used the following antibodies: pERK1/2 (cat#9101, Cell Signaling, USA), total-ERK1/2, (cat#9102, Cell Signaling, USA), and beta-actin or tubulin (Sigma, USA).

### Cell invasion and migration assays

Cell invasion assays were performed using 24-well BioCoat Matrigel invasion chambers according to manufacturer's instructions (BD Biosciences, USA). These chambers were used to study the effect of vemurafenib or vehicle on the invasion of PTC or NT cells (8×10^3^ or 25×10^3^ cells/assay) for about 20 hours in culture. Invasion assay was performed in a serum free growth medium and we used 5% FBS as chemo-attractant agent. Migration assay was performed using: 3×10^3^ or 25×10^3^ PTC or NT cells/assay, 6×10^3^ or about 25×10^3^ pericytes/assay, 30-100×10^3^ BEC or LEC/assay loaded into the migration chamber (Corning Incorporated, Corning, NY, USA) and grown for a range of 3-12 hours in culture. PTC or NT cell migration assay was performed in a serum free growth medium and we used 5% FBS as chemo-attractant agent. The secreted pro-angiogenic or anti-angiogenic factors (secretome) derived from the human PTC with or without *BRAF^WT/V600E^* or from NT cells treated with vehicle (2% DMSO) or vemurafenib (10 μM) for 24 hours was utilized as chemo-attractant conditioning medium (CM) to induce endothelial cell migration (Supplementary Figure 4A). Migrated or invaded cells were quantified in all assays as described: cells were counted (number of cells/field or number of cells/well) using a 10× or 20× objective, and four fields were chosen per well with two well per each condition.

### *In vitro* angiogenesis assay

*In vitro* angiogenesis assays were performed as previously described [48] [46]. In brief, BEC or LEC (40-80×10^3^ cells) (with starvation overnight at 1% serum) were suspended in NT or PTC-derived secretome treated with vehicle or vemurafenib (10 μM) for 24 hours and seeded on growth factors-reduced Matrigel (cat#354230, BD Biosciences, USA) in 24-well petri dishes. The secretome derived from the PTC or NT cells was utilized to induce tube (tubule)-like structures formation (Supplementary Figure 4B). After about 6-8 hours of incubation, cells were photographed. The number of tubes was counted using a 10× or 20× objective and four fields were chosen per well with two wells per each condition. Human Leptin blockage was performed using an antibody against Leptin (cat#AF398, R&D Systems, MN, USA).

### Quantitative multigene profiling mRNA expression analysis

The expression of EMC and angiogenesis genes was validated by multi-gene transcriptional profiles provide a quantitative view of the expression of many genes [49]. Cells were seeded at about 60-80% confluence in 6-well dishes and grown in 1:1 DMEM/Ham's F12 or high glucose DMEM supplemented with 0.2% FBS. After 24 hours, RNA isolation was performed by Quiagen columns (Quiagen, USA) following manufacturer protocols. Quantitative multi-gene profiling was performed by absolute quantification using real-time reverse transcriptase PCR (RT-PCR) according to Shih et al. [49]. Primer sequences used for the validation of human genes are reported in Supplementary Table 6. We classified gene expression as ‘low copy number’ if it was below 1 mRNA copy/10^6^ 18S copies, ‘moderate copy number’ if it was between 1 and 15 mRNA copies/10^6^ 18S copies, and ‘high copy number’ if it was greater than 15 mRNA copies/10^6^ 18S copies. Genes showing differences in relative values of comparisons (p values <0.05) were considered differentially expressed.

### Orthotopic mouse model

All animal work was done in accordance with federal, local, and institutional guidelines at the Beth Israel Deaconess Medical Center (Boston, MA, USA). Human metastatic KTC1 tumor-derived cells harboring the heterozygous BRAF^V600E^ mutation engineered to express GFP were cultured in 10-cm dishes and grown in high glucose DMEM medium supplemented with 10% FBS, penicillin, streptomycin, and amphotericin at 37°C with 5% CO_2_ atmosphere. On the day of tumor implantation, the cells were trypsinized, gently centrifuged, and suspended in serum-free high glucose DMEM growth medium to achieve a cell suspension concentration ranged between 3×10^6^ cells and 5×10^6^ cells/10 μL. The cells were kept on ice until implantation. KTC1 cells were orthotopically injected using a Hamilton syringe (Fischer, MA, USA) in the right thyroid of 9 weeks old male NOD SCID gamma mice (strain name: NOD.Cg-Prkdc^scid^ Il2rg^tm1Wjl^/SzJ; stock number: 005557) (The Jackson Laboratory, Bar Harbor, Maine, USA) (BIDMC IRB, Boston) (n=5 per group) according to our previous experimental procedures [1, 50, 51]. Mice were randomly divided into two equal groups of 5 mice for the purpose of establishing a time course of tumor cell growth and response to the early therapeutic intervention with vemurafenib. Vemurafenib treatment was started 6 days (early intervention trial) after KTC1 tumor cells implantation and performed for about 12 days.

### Apoptosis assay *in vivo*

Apoptosis assay was performed using terminal deoxynucleotidyl transferase dUTP nick end labeling (TUNEL) assay according to manufacturer recommendation (ApopTag peroxidase *in situ* detection kit) (Millipore Corp., Bedford, MA); apoptotic cells were assessed semi-quantitatively using the following scoring method: 0 negative, 1, 1–10% positive cells, 2, 11–50% positive cells, and 3, more than 50% positive cells.

### Statistical analysis

Statistical analysis was carried out using GraphPad Prism 6 software (San Diego, CA, USA) and statistical tools by Microsoft Excel 97-2003 (Boston, MA, USA). Mann-Whitney, one-way ANOVA, T-student and Fisher's exact tests were used. Data are reported as the averaged value, and error bars represent the standard deviation or standard error mean of the average for each group. Results with *p* values below 0.05 were considered statistically significant.

## SUPPLEMENTARY RESULTS FIGURES AND TABLES








