# Supplementary material for: Metastasis-associated MCL1 and P16 copy number alterations dictate resistance to vemurafenib in a BRAFV600E patient-derived papillary thyroid carcinoma preclinical model
Source: Oncotarget. 2015 Nov 30;6(40):42445–67. doi: 10.18632/oncotarget.6442 (PMC4767444; doi:10.18632/oncotarget.6442)
Supplement: Supplementary file 1 [file oncotarget-06-42445-s001.pdf]

Metastasis-associated *MCL1* and *P16* copy number alterations dictate resistance to vemurafenib in a *BRAF*^{V600E} patient-derived papillary thyroid carcinoma preclinical model

Supplementary Results

Effects of anti-BRAF^{V600E} therapy *in vivo*

Vemurafenib therapy significantly (p=0.007) reduced 2-fold vascular density in primary orthotopic *BRAF*$^{WT/V600E}$-KTC1 tumor compared with control (vehicle-treated) orthotopic BRAF^{V600E}-tumor mice **(Figure 2E)**. Moreover, we found that nuclear phospho(p)-AKT protein levels were substantially decreased in the vemurafenib-treated tumors compared with the vehicle-treated orthotopic *BRAF*$^{WT/V600E}$-tumors **(Figure 2F)**. We also analyzed protein expression of different molecules (cyclin E1, cyclin E2, cyclin B2 and p27/kip1) involved in cell-cycle checkpoints and cell proliferation. Importantly, we found that vemurafenib substantially inhibited cell cycle progression by down-regulating nuclear protein levels of cyclin B2, from about 30% of tumor cells in the vehicle-treated tumors showing cyclin B2 expression to about 2% of tumor cells in vemurafenib-treated tumors **(Figure 2F)**. We also assessed pro-inflammatory/stromal biomarkers, such as CD45 (leucocyte marker) and F4-80 (macrophage marker (**Figure 2I)**, and PDGFRB (pericyte lineage and tumor cell marker, **Figure 2I**), which might contribute to the pro-invasive behavior of PTC cells by increasing extracellular matrix (ECM) molecule levels in the orthotopic tumor microenvironment. Vemurafenib treatment did not substantially reduce the numbers of lymphocytes or macrophages and did not affect the protein levels of these markers, with the exception of PDGFRB. PDGFRB exhibited statistically significant (2.2-fold, p=0.007) higher protein levels (diffuse and strong staining) in the peri-tumor and intra-tumor areas of the orthotopic *BRAF*$^{WT/V600E}$-tumor in mice treated with vehicle (control) compared with vemurafenib-treated orthotopic *BRAF*$^{WT/V600E}$-tumor mice (focal

staining) **(Figure 2I)**, suggesting a synergistic biological role between the PDGFRB-positive stromal cells (i.e. pericytes) and *BRAF*$^{WT/V600E}$-positive tumor cells which ultimately might trigger angiogenesis and angioinvasion.

BRAF$^{WT/V600E}$-PTC cells recruit endothelial cells and pericytes by regulating pro-angiogenic/metastatic paracrine signaling

We found that both LEC and BEC migration was significantly higher in the presence of primary metastatic/recurrent *BRAF*$^{WT/V600E}$-PTC cell-derived secretome compared with migration of endothelial cells grown in the presence of secretome derived from non-metastatic *BRAF*$^{WT/V600E}$-PTC cells (for LEC= 1.6-fold increase, p=0.02; for BEC= 1.4-fold increase, p=0.02), *BRAF*^{WT}-PTC cells (for LEC= 2.1-fold increase, p=0.02; for BEC= 3.3-fold increase, p=0.02) or primary NT cells (for LEC= 10.8-fold increase, p=0.02; for BEC= 19.8-fold increase, p=0.02) **(Suppl. Figure 4E)**.

Furthermore, we analyzed the effects of *BRAF*$^{WT/V600E}$-PTC cells on pericytes functions because of their fundamental role to stabilize vessels and regulate angiogenesis. Pericytes migration **(Suppl. Figure 4C)** was also significantly higher in the presence of vehicle-treated secretome derived from primary *BRAF*$^{WT/V600E}$-positive metastatic/recurrent PTC cells (which did not induce pericytes tubules formation *in vitro*, **Suppl. Figure 4D)** compared with migration of pericytes grown in the presence of secretome derived from *BRAF*^{WT}-PTC cells (1.6-fold increase, p=0.02) or primary NT cells (4.2-fold increase, p=0.02). Importantly, non-metastatic *BRAF*$^{WT/V600E}$-PTC-derived vemurafenib-treated secretome significantly suppressed *in vitro* migration of pericyte (p=0.02) **(Suppl. Figure 4C)**, LEC (p=0.02) and BEC (p=0.02) **(Suppl. Figure 4E and 4H)** compared to secretome vehicle-treated. As a result of significant LEC and

BEC migration *in vitro*, these endothelial cells formed tubules in a reconstituted ECM basement membrane (Matrigel) when stimulated by non-metastatic *BRAF*$^{WT/V600E}$-PTC cell–derived secretome, and the rate of tubule formation was significantly more rapid than the same cells grown in the presence of secretome derived from *BRAF*^{WT}-PTC cells (for LEC=1.2-fold increase, p=0.02; for BEC= 1.3-fold increase, p=0.03) or NT cells (for LEC=2.4-fold increase, p=0.02; for BEC= 2.8-fold increase, p=0.01) **(Figure 3A** and **Suppl. Figure 4F)** or compared with the growth medium. Metastatic/recurrent *BRAF*$^{WT/V600E}$-PTC short-term cell cultures– **(Figure 3A)** or spontaneously immortalized human PTC cell-derived secretome was even more potent in inducing either LEC or BEC tubule formation **(Suppl. Figure 4G)** compared to secretome derived from non-metastatic *BRAF*$^{WT/V600E}$-PTC cells (for LEC=1.3-fold increase, p=0.03; for BEC= 1.2-fold increase, p=0.03), *BRAF*^{WT}-PTC cells (for LEC=1.6-fold increase, p=0.01; for BEC= 1.6-fold increase, p=0.03) or NT cells (for LEC=3.3-fold increase, p=0.03; for BEC= 3.4-fold increase, p=0.01) **(Figure 3A)**.

We also sought to determine the angiogenic microenvironment effectors of BRAF$^{WT/V600E}$ and used a multiplex ELISA assay that included the most known pro-angiogenic and anti-angiogenic factors. BRAF^{V600E} dictated protein expression of pro-angiogenic factors and ECM molecules in human PTC cells. In details, we found that LN metastatic/recurrent *BRAF*$^{WT/V600E}$-PTC cells secreted significantly higher protein levels of the pro-angiogenic factor Leptin (~2-fold) *in vitro* compared with *BRAF*^{WT}-PTC cells (p=0.01) or NT cells (p=0.01) **(Figure 3B)**. Interestingly, metastatic/recurrent *BRAF*$^{WT/V600E}$-PTC produced and secreted significantly higher Leptin protein compared to secreted VEGFA (186-fold increase, p=0.0001) or TSP-1 (Thrombospondin-1) protein levels (118-fold increase, p=0.0001) **(Figure 3B)**. Inhibition of BRAF$^{WT/V600E}$ kinase with vemurafenib in metastatic/recurrent *BRAF*$^{WT/V600E}$-PTC cells

significantly diminished: (i) Leptin protein levels (p=0.02) **(Figure 3C)** (but not mRNA expression levels, **Suppl. Table 4**), and (ii) VEGFA (p=0.03) and TSP-1 (p=0.01) protein and mRNA expression levels compared to vehicle-treated *BRAF*$^{WT/V600E}$-PTC cells **(Figure 3B** and **Suppl. Table 4)**. Mechanistically, we found that the use of a human Leptin blocking antibody statistically significant suppressed LEC (2.6-fold decrease, p=0.02) and BEC (3-fold decrease, p=0.02) microvascular endothelial tubules formation **(Suppl. Figure 4I-J)** in the presence of vehicle-treated secretome derived from metastatic/recurrent *BRAF*$^{WT/V600E}$-PTC cells. The rate of tubule suppression by the human Leptin blocking antibody was even more potent (4.3-fold decrease, p=0.02) when BEC were grown in the presence of vemurafenib-treated secretome derived from primary metastatic/recurrent *BRAF*$^{WT/V600E}$-PTC cells **(Suppl. Figure 4J)**, suggesting that inhibition of both BRAF^{V600E} and Leptin pathways was more effective to reduce angiogenesis *in vitro*. No statistically significant changes in tubules formation were observed in LEC or BEC grown in the presence of vehicle-treated or vemurafenib-treated secretome derived from *BRAF*^{WT}-PTC cells or NT cells in the presence or not of Leptin blocking antibody.

Understanding the extent to which tumor microenvironment factors participate in the aberrant behavior of *BRAF*^{V600E}-positive metastatic PTC cells will reveal whether the microenvironment is a promising target for development of new therapies. Here, our translational PTC model revealed that metastatic/recurrent *BRAF*^{V600E}-PTC clinical samples statistically significant (p=0.02) showed 2.8-fold higher PDGFRB protein expression levels compared to *BRAF*^{WT}-PTC **(Figure 3C)**. To examine this result more in details, we used co-localization approach and co-stained for PDGFRB and PAX8 (thyrocyte cell marker); we found that PDGFRB protein levels were significantly higher (2.2-fold increase, p=0.02) in metastatic *BRAF*^{V600E}-PTC-associated stromal cells (i.e. mesenchymal stem cell–like pericytes) compared to

PAX8-labeled metastatic *BRAF*^{V600E}-PTC cells in the tumor microenvironment **(Figure 3C)**. Furthermore, *BRAF*^{V600E}-PTC samples showed statistically significant higher PDGFRB protein levels in both stromal cells and PTC cells as compared to *BRAF*^{WT}-PTC samples (*BRAF*^{WT} PTC cells=2.5-fold decrease, p=0.02; *BRAF*^{WT} PTC associated stromal cells=1.5-fold decrease, p=0.02) **(Figure 3C)**. It is possible that pericytes synergize with adjacent human microvascular endothelial cells and prime PTC microenvironment to accommodate tumor cell migration by hyper-secreting ECM molecules. Therefore, we also interrogated the role of PTC cells on endothelial cell function using PTC formalin fixed paraffin-embedded clinical samples. Similarly to our *in vitro* results **(Figure 3A)**, we found that *BRAF*^{V600E}-positive PTC statistically significant showed intra- and peri-tumor blood and lymphatic vessels at 2-fold (p=0.02) and 6-fold (p=0.02) higher rates, respectively, than *BRAF*^{WT}-PTC cells (p<0.02) **(Figure 3C** and **Suppl. Figure 5)**.

Vemurafenib therapy prevents migration and invasion in non-metastatic *BRAF*$^{WT/V600E}$-PTC cells

Consistently with the ability of metastatic primary *BRAF*$^{WT/V600E}$-PTC cells to trigger angiogenesis *in vitro* **(Figure 3A, Suppl. Figure 4G)** and endothelial cell migration **(Suppl. Figure 4E and Suppl. Figure 4H)**, they also showed a statistically higher tendency to migrate (mean= 15.6 migrated PTC cells/transwell, SD=0.5, p=0.01) or invade (mean= 17.6 invaded PTC cells/transwell, SD=0.5, p=0.01) through Matrigel *in vitro* **(Figure 3D)**. Results of *BRAF*$^{WT/V600E}$-PTC derived spontaneously immortalized KTC1 and BCPAP cells migration and invasion are reported in **Suppl. Figure 6A-B** as compared with non-metastatic *BRAF*^{V600E}-PTC cells (mean=11.3 migrated cells/transwell, SD=0.5, p=0.01; mean=10 invaded cells/transwell, SD=1, p=0.03) (**Figure 3D**), and *BRAF*^{WT}-PTC cells (mean=8 migrated cells/transwell, SD=1,

p=0.03; mean=7.6 invaded cells/transwell, SD=0.5, p=0.02) or NT cells (mean= 1.6 migrated cells/transwell, SD=0.5, p=0.01; mean=2 invaded cells/transwell, SD=1, p=0.01) **(Figure 3D)**. Vemurafenib therapy statistically significant (p=0.01) reduced cell migration and invasiveness by approximately 3.5-fold in non-metastatic *BRAF*$^{WT/V600E}$-PTC cells *in vitro* as compared with vehicle-treated (control) cells **(Figure 3D**).

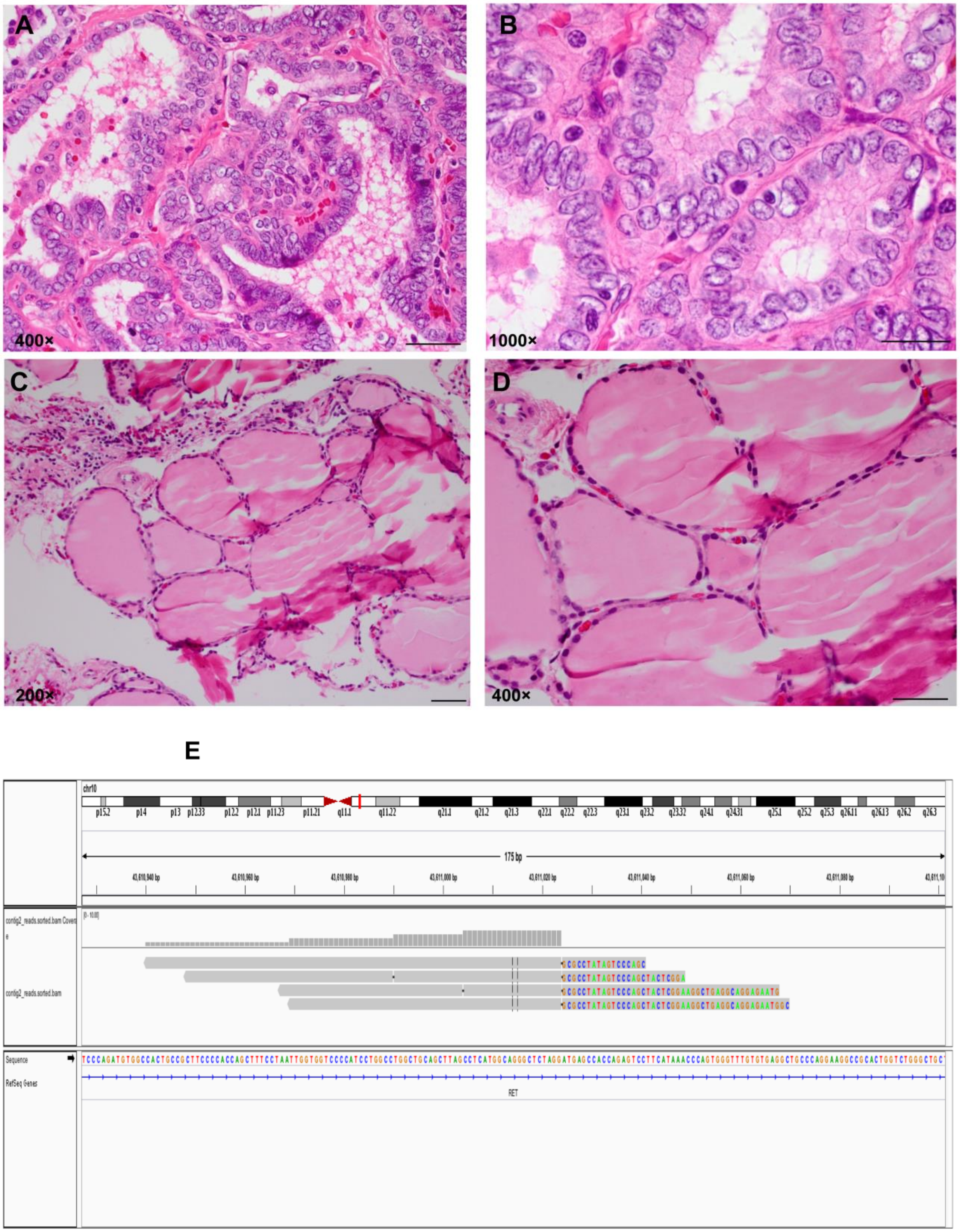
A
400×
B
1000×
C
200×
D
400×
E
chr10
175 bp
Sequence
RefSeq Genes
RET

Suppl. Figure 1

A) Hematoxylin and eosin (HE)-stained formalin-fixed paraffin-embedded (FFPE) classical papillary thyroid carcinoma (PTC) tissue. Scale bars are=400 µ.

B) High magnification photos of classical PTC showing nuclear clearing. Scale bars are=500 µ.

C-D) Human normal thyroid (NT) tissue specimen. Scale bars are=200 µ (200× magnification) and 400 µ (400× magnification).

E) Translocation(rearrangement) analysis in human papillary thyroid carcinoma (PTC) cells *in vitro*. Next generation sequencing reads that partially aligned to the targeted regions captured and sequenced. The unaligned portion of these 'soft-clipped' reads was used to assemble contigs. The contigs were aligned to the reference genome, and structural variants were called based on the alignment. For translocation events, discordantly mapped paired-end reads were identified which flank putative breakpoints. A well-known translocation event was detected in a PTC sample with no $BRAF^{V600E}$ mutation (but with $BRAF^{WT/WT}$) between genes *RET-PTC3* (NCOA4) on chromosome 10. There were four split-reads and 6 discordantly mapped read-pairs supporting the translocation event.

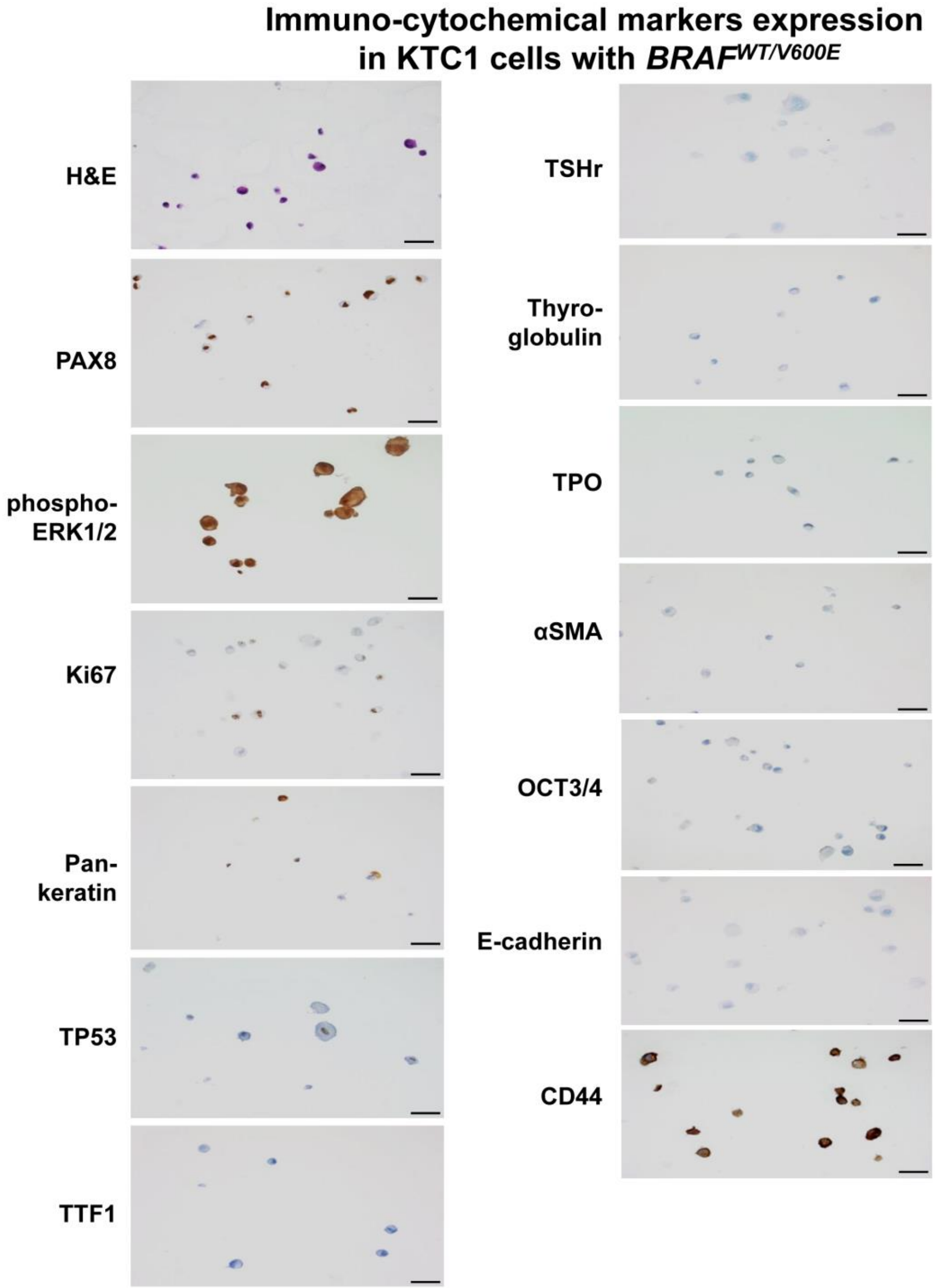

Suppl. Figure 2

Immunocytochemistry analysis of biomarkers in human metastatic papillary thyroid carcinoma (PTC)-derived KTC1 cells with *BRAF*$^{WT/V600E}$.

Hematoxylin and eosin (HE)-stained sections of formalin-fixed paraffin-embedded (FFPE) cell blocks. Immunohistochemical analysis of different markers in KTC1 cells, a spontaneously immortalized metastatic *BRAF*$^{WT/V600E}$-PTC cell shows strong protein expression for: PAX8, phospho(p)-ERK1/2, ki67, pan-keratin and CD44; and weak/focal protein expression for TP53. Magnifications: 400×. All scale bars are=100 µ.

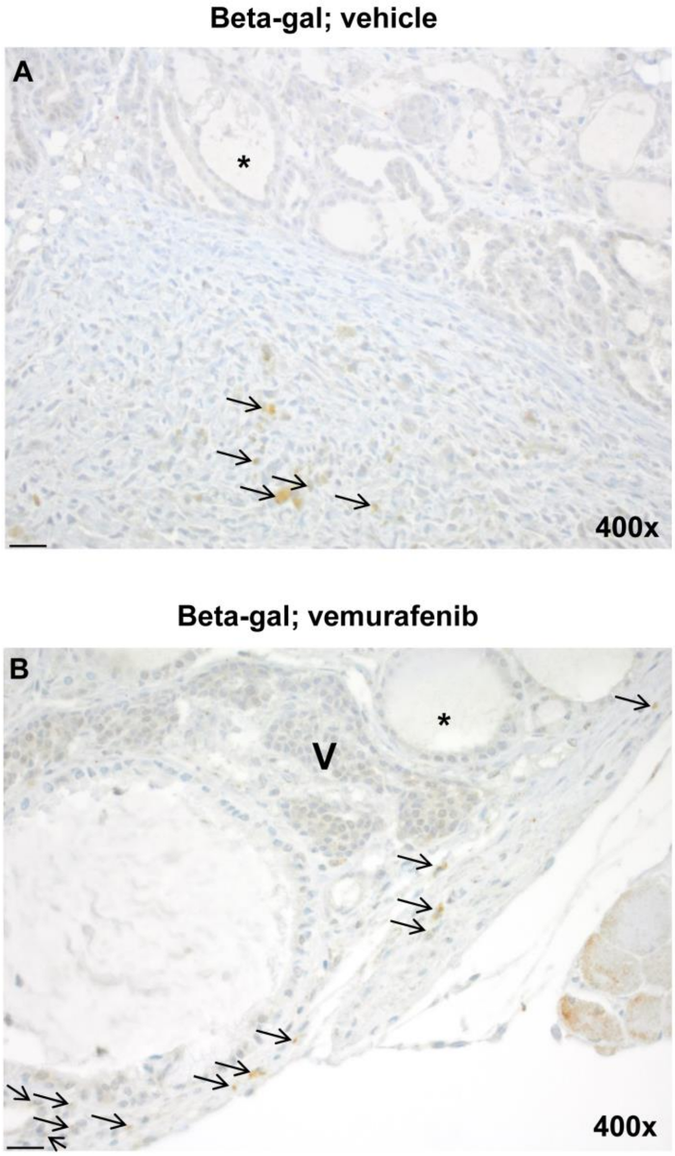

Suppl. Figure 3

Immunohistochemistry analysis of beta-galactosidase (β-gal) expression in the human KTC1 orthotopic tumor with $\mathit{BRAF}^{WT/V600E}$.

Formalin-fixed paraffin-embedded (FFPE) sections of orthotopic human papillary thyroid cancer (PTC)-derived cells harboring the heterozygous $\mathit{BRAF}^{WT/V600E}$ mutation, with homozygous loss of *P16* using vemurafenib in 9 weeks old male NOD SCID immunocompromised (gamma) (strain name: NOD.Cg-Prkdc^{scid} $\mathrm{Il2rg}^{tm1Wjl}$/SzJ) mice (n=10). Immunohistochemical analysis shows human lysosomal endogenous beta-galactosidase (β-gal) expression in the vehicle-treated orthotopic $\mathit{BRAF}^{WT/V600E}$ KTC1 cells (arrows, **A**) compared with vemurafenib-treated orthotopic $\mathit{BRAF}^{WT/V600E}$ KTC1 PTC (arrows, **B**) at day 18. Asterisk highlights mouse normal thyroid follicle and V highlights mouse parathyroid. Magnifications: 400×. All scale bars are=400 µ.

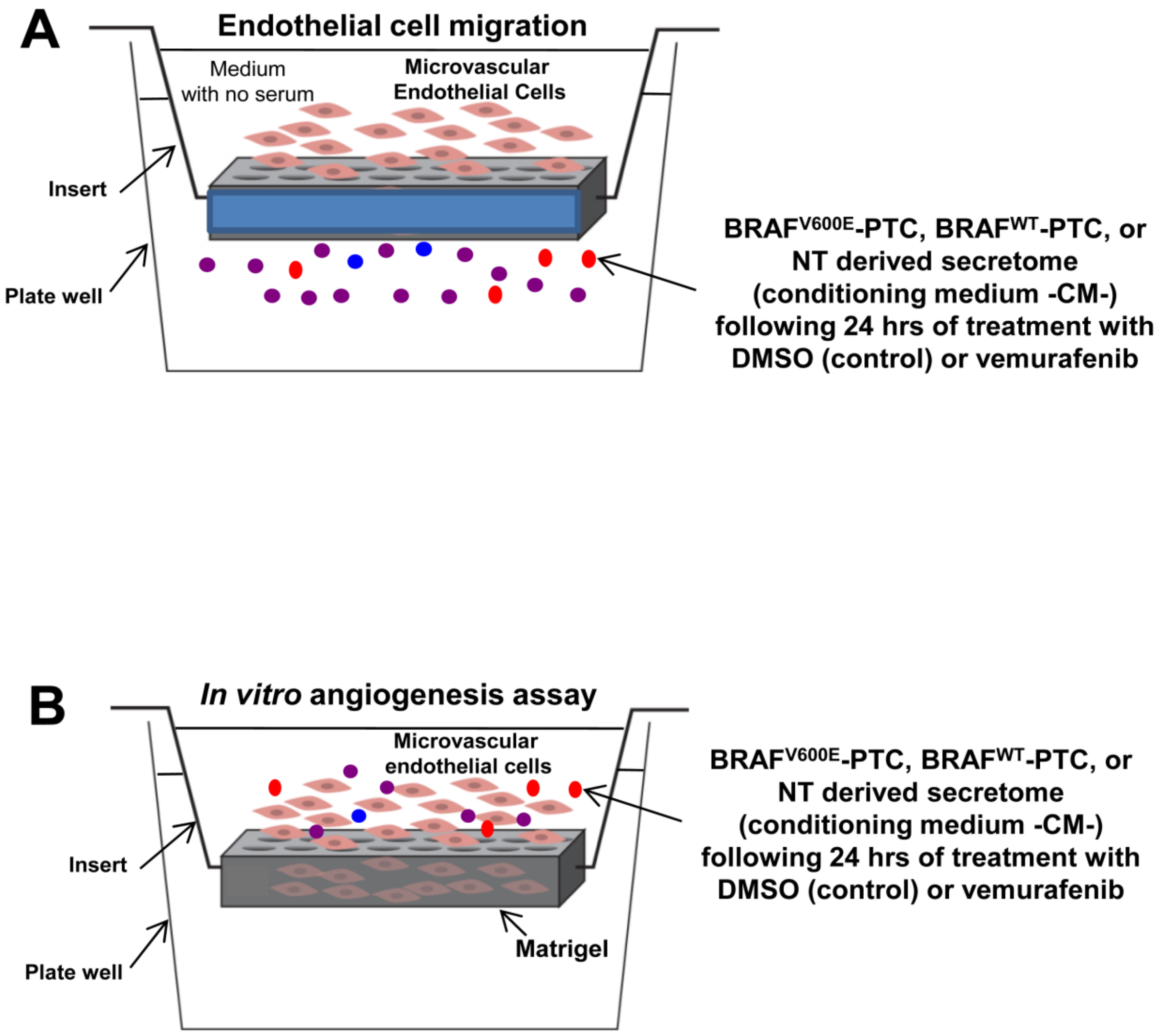
A
Endothelial cell migration
Medium
with no serum
Microvascular
Endothelial Cells
Insert
Plate well
BRAF^{V600E}-PTC, BRAF^{WT}-PTC, or
NT derived secretome
(conditioning medium -CM-)
following 24 hrs of treatment with
DMSO (control) or vemurafenib
B
In vitro angiogenesis assay
Microvascular
endothelial cells
Insert
Plate well
Matrigel
BRAF^{V600E}-PTC, BRAF^{WT}-PTC, or
NT derived secretome
(conditioning medium -CM-)
following 24 hrs of treatment with
DMSO (control) or vemurafenib

Human pericytes migration assay

C

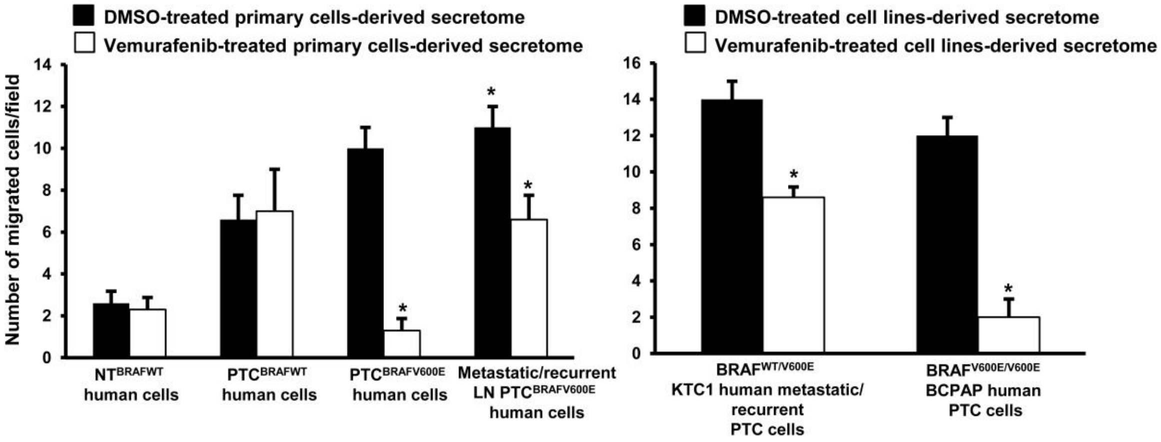

D

Matrigel assay (tubule formation)

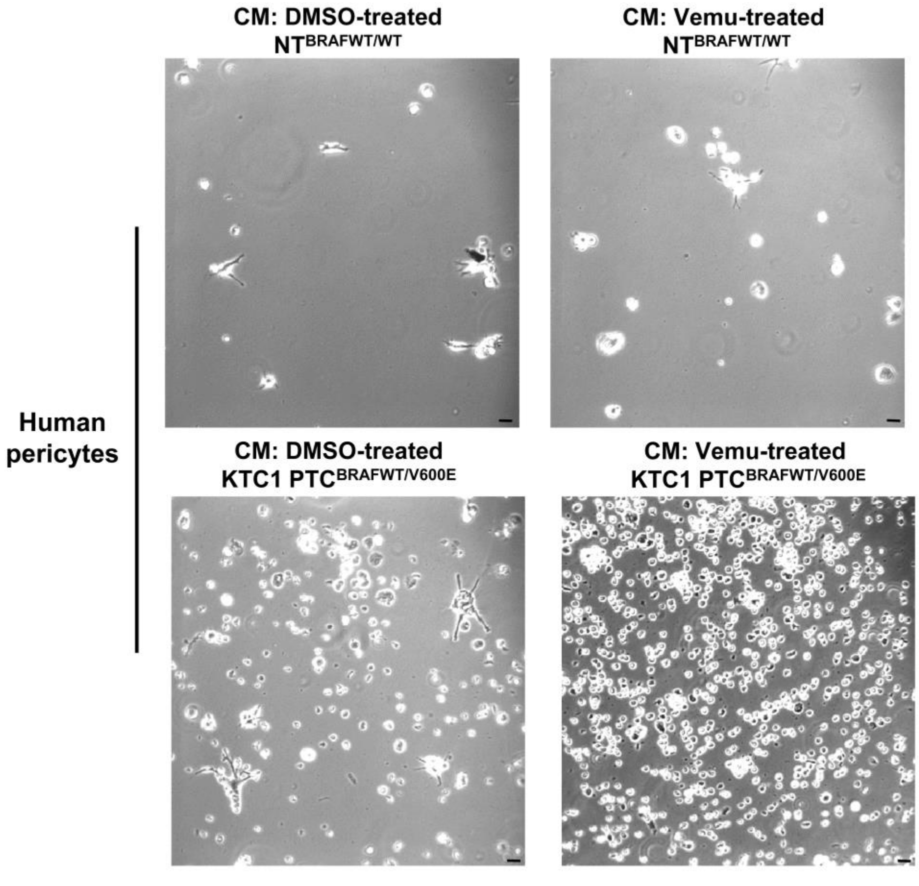

E

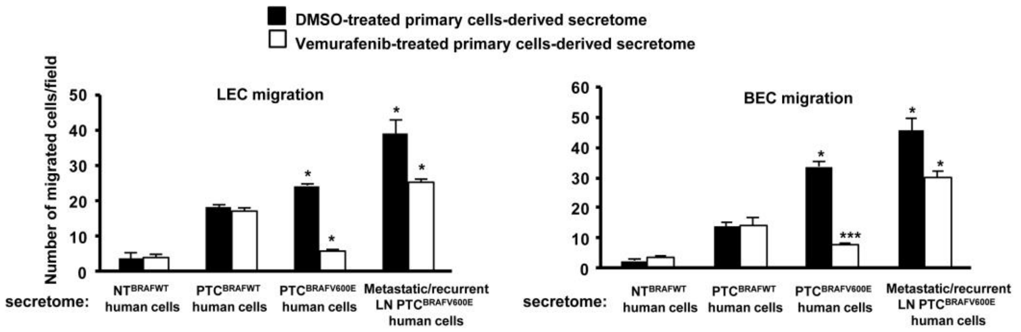
DMSO-treated primary cells-derived secretome
Vemurafenib-treated primary cells-derived secretome
LEC migration
BEC migration
Number of migrated cells/field
0
10
20
30
40
50
60
*
***
secretome:
NT^BRAFWT human cells
PTC^BRAFWT human cells
PTC^BRAFV600E human cells
Metastatic/recurrent LN PTC^BRAFV600E human cells

F

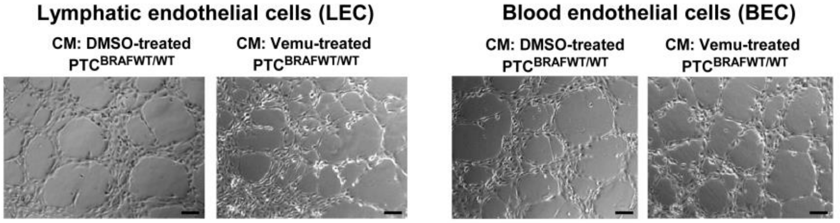
Lymphatic endothelial cells (LEC)
Blood endothelial cells (BEC)
CM: DMSO-treated PTC^BRAFWT/WT
CM: Vemu-treated PTC^BRAFWT/WT

G

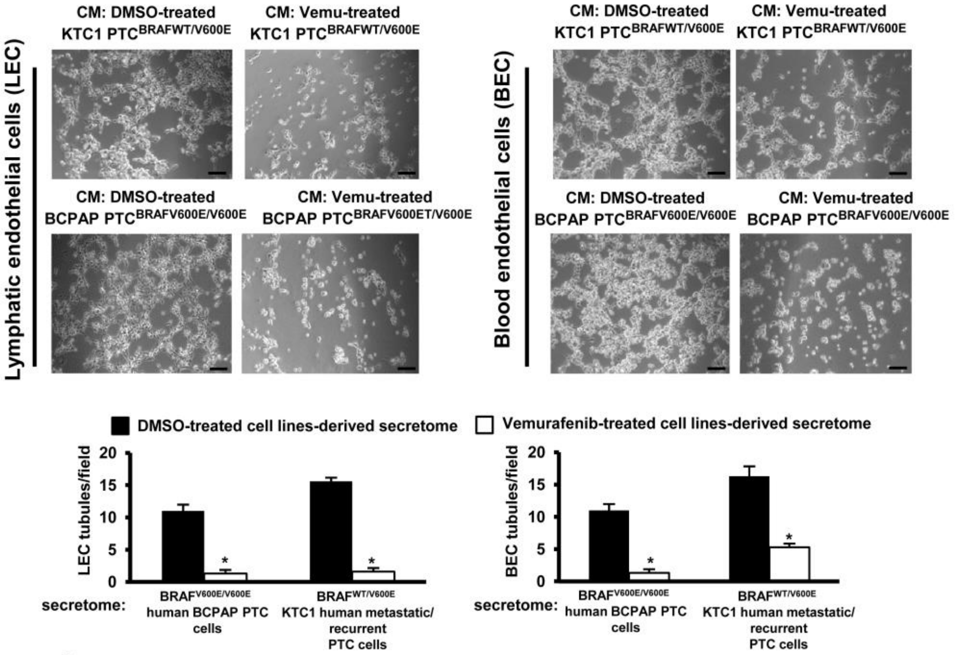
Lymphatic endothelial cells (LEC)
Blood endothelial cells (BEC)
CM: DMSO-treated KTC1 PTC^BRAFWT/V600E
CM: Vemu-treated KTC1 PTC^BRAFWT/V600E
CM: DMSO-treated BCPAP PTC^BRAFV600E/V600E
CM: Vemu-treated BCPAP PTC^BRAFV600ET/V600E
CM: Vemu-treated BCPAP PTC^BRAFV600E/V600E
DMSO-treated cell lines-derived secretome
Vemurafenib-treated cell lines-derived secretome
LEC tubules/field
BEC tubules/field
0
5
10
15
20
*
secretome:
BRAF^V600E/V600E human BCPAP PTC cells
BRAF^WT/V600E KTC1 human metastatic/ recurrent PTC cells

H

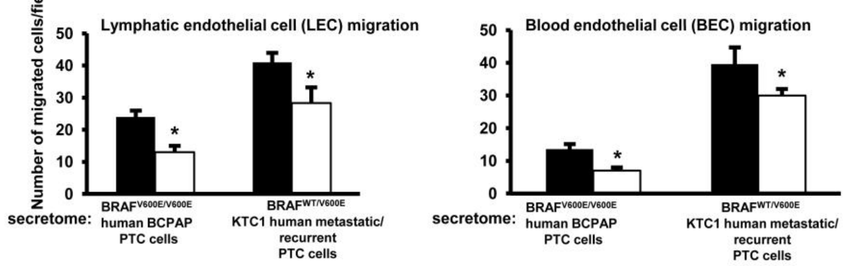
Lymphatic endothelial cell (LEC) migration
Blood endothelial cell (BEC) migration
Number of migrated cells/field
0
10
20
30
40
50
*
secretome:
BRAF^V600E/V600E human BCPAP PTC cells
BRAF^WT/V600E KTC1 human metastatic/ recurrent PTC cells

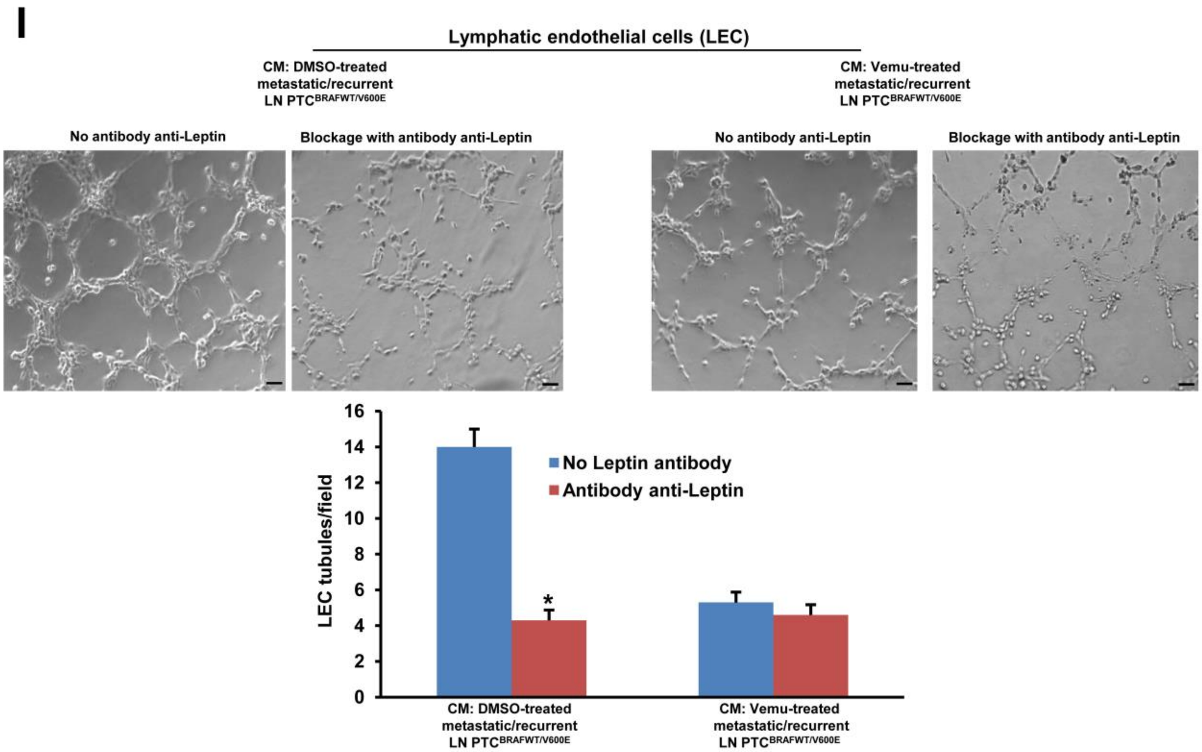

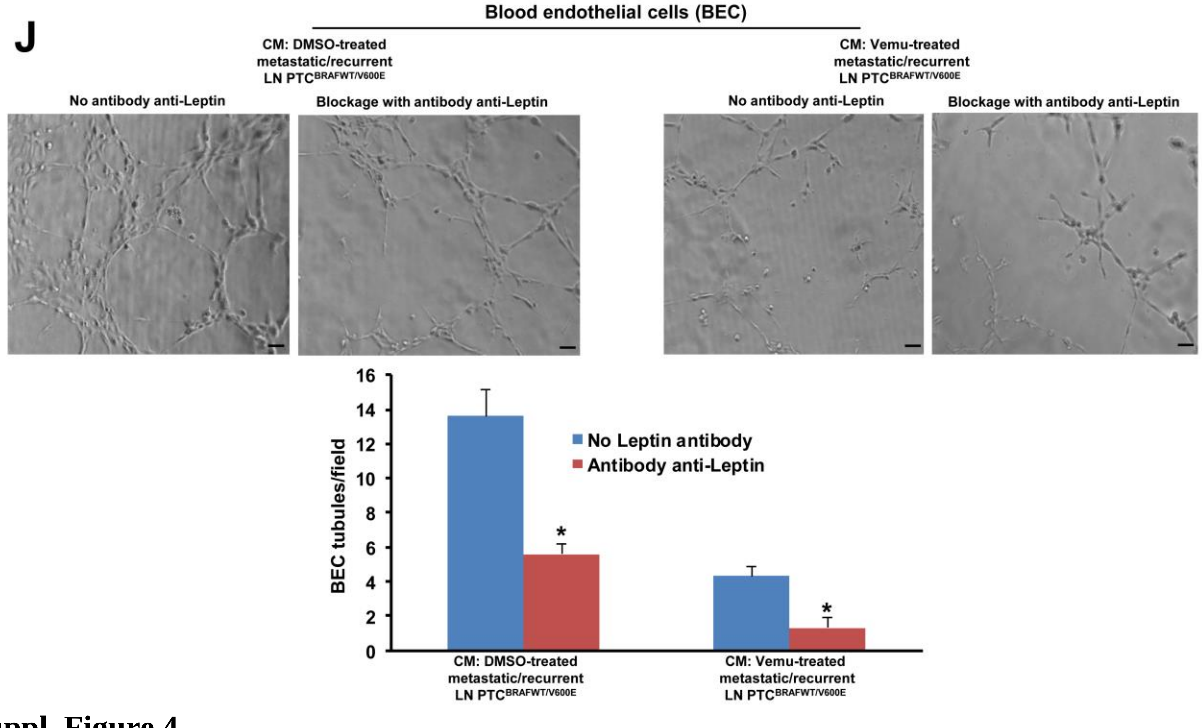

Suppl. Figure 4
***BRAF*^{V600E}-positive human papillary thyroid carcinoma (PTC) cell promotes human microvascular endothelial cell migration and angiogenesis *in vitro* and pericyte migration *in vitro*.**

A) *In vitro* angiogenesis (tube formation) assay: human blood and lymphatic microvascular endothelial cells (BEC and LEC, respectively) were suspended in human NT or PTC-derived secretome treated with vehicle or vemurafenib (10 µM) and seeded on growth-factor-reduced Matrigel. The secretome derived from the PTC or NT cells was utilized to induce tube formation. After about 5-6 hours of incubation, cells were photographed. The number of tubes was counted using a 10× or 20× objective in four random fields or per well with two wells per each condition.
B) Migration assay: human blood and lymphatic microvascular endothelial cells (BEC and LEC, respectively) were loaded into the migration chamber and grown for 3-5 hours in culture. The secreted pro-angiogenic/growth or anti-angiogenic factors (secretome) treated derived from human PTC cells with or without $BRAF^{V600E}$, or from NT cells with treated with vehicle (DMSO) or 10 µM vemurafenib were utilized as chemo-attractant conditioning medium (CM) to induce cell migration. Migrated cells were quantified as number of cells/field or number of cells/well using a 10× or 20× objective, and four fields were chosen per well.
C) Pericyte migration *in vitro:* the secreted pro-angiogenic/growth or anti-angiogenic factors (secretome) derived from human primary PTC cells with or without $BRAF^{V600E}$, from spontaneously immortalized human PTC-derived BCPAP cells (homozygous $BRAF^{V600E}$), metastatic PTC-derived KTC1 cells (heterozygous $BRAF^{V600E}$), or NT primary cells were utilized as chemo-attractant conditioning medium to induce cell migration for about 5 hours in culture.
These data represent the average ± standard deviation (error bars) of 2 independent experiments replicate measurements (*p<0.05, Mann-Whitney test).
D) Pericytes and angiogenesis *in vitro* assay: metastatic PTC-derived spontaneously immortalized human KTC1 cells (heterozygous $BRAF^{V600E}$) or primary NT cells derived secretome do not trigger pericyte tube formation *in vitro*. Pericytes were suspended in primary NT cells or KTC1 cells-derived secretome treated with vehicle or vemurafenib (10 µM) for 24 hours and seeded on growth-factor-reduced Matrigel. The secretome was utilized to induce microvascular endothelial cell tube formation *in vitro*. No tube formation and no significant changes were observed in any experimental conditions. CM= conditioning medium. Magnification: 10×. All scale bars are=50 µ. These data represent 2 independent experiments replicate measurements.
E) Human blood and lymphatic microvascular endothelial cell (BEC and LEC, respectively) migration assays: LEC and BEC migration assay was performed for about 5 hours in culture. The secreted pro-angiogenic/growth or anti-angiogenic factors (secretome) derived from PTC cells with or without $BRAF^{WT/V600E}$ or from NT cells was utilized as chemo-attractant conditioning medium to induce cell migration. These data represent the average ± standard deviation (error bars) of 2 independent experiments replicate measurements (*p<0.05, Mann-Whitney test).
F) Angiogenesis *in vitro* (microvascular endothelial cell tube formation): $BRAF^{WT}$-human PTC-derived secretome triggers human lymphatic and blood microvascular endothelial cell (LEC and BEC, respectively) tube formation *in vitro*. LEC and BEC were suspended in NT or PTC-derived secretome (conditioning medium (CM)) treated with vehicle or vemurafenib (10 µM) for 24 hours and seeded on reduced-growth-factor Matrigel. The secretome was utilized to induce tube formation. LEC and BEC were photographed after 5-6 hours. Images show no significant changes in either LEC or BEC tubes formation in the presence of secretome derived from PTC cells with $BRAF^{WT}$. Magnification: 10×. All scale bars are=200 µ. These data represent 2 independent experiments replicate experiments.

G) Angiogenesis *in vitro* (microvascular endothelial cell tube formation): KTC1 (human spontaneously immortalized heterozygous *BRAF*$^{WT/V600E}$-PTC) or BCPAP (human spontaneously immortalized homozygous *BRAF*$^{V600E/V600E}$-PTC) cell-derived secretome triggers LEC and BEC tube formation *in vitro*. LEC and BEC were suspended in KTC1-derived secretome (conditioning medium (CM)) treated with vehicle or vemurafenib (10 µM) for 24 hours and seeded on growth-factor-reduced Matrigel. The secretome was utilized to induce microvascular endothelial cell tube formation *in vitro*. LEC and BEC tended to form short and thick tubes with very condensed clusters of cells (photographed after 5-6 hours). Histograms show significant changes in both LEC and BEC tubules formation in the presence of DMSO (control)-treated secretome vs. vemurafenib-treated secretome. Magnification: 10×. All scale bars are=200 µ. These data represent the average ± standard deviation (error bars) of 2 independent experiments replicate measurements (*p<0.05, Mann-Whitney test).
H) Human microvascular endothelial cell migration assays in the presence of DMSO (control)-treated or vemurafenib-treated secretome derived from human spontaneously immortalized heterozygous *BRAF*$^{WT/V600E}$-PTC KTC1 cells or human spontaneously immortalized homozygous *BRAF*$^{V600E/V600E}$-PTC BCPAP cells. LEC and BEC migration assay was performed for about 5 hours. The secreted pro-angiogenic or anti-angiogenic factors (secretome) derived from BCPAP or KTC1 PTC cells was utilized as chemo-attractant conditioning medium to induce cell migration. These data represent the average ± standard deviation (error bars) of 2 independent experiments replicate measurements (*p<0.05, Mann-Whitney test).
I-J) Blockage of human Leptin activity by using a neutralizing antibody. The use of human Leptin blocking antibody efficiently reduces human blood and lymphatic microvascular endothelial cell (BEC and LEC, respectively) tubules formation in the presence of metastatic/recurrent *BRAF*$^{WT/V600E}$-PTC cell–derived vehicle-treated or vemurafenib-treated secretome. Magnification: 10×. All scale bars are=200 µ. These data represent the average ± standard deviation (error bars) of independent replicate measurements (*p<0.05, Mann-Whitney test).

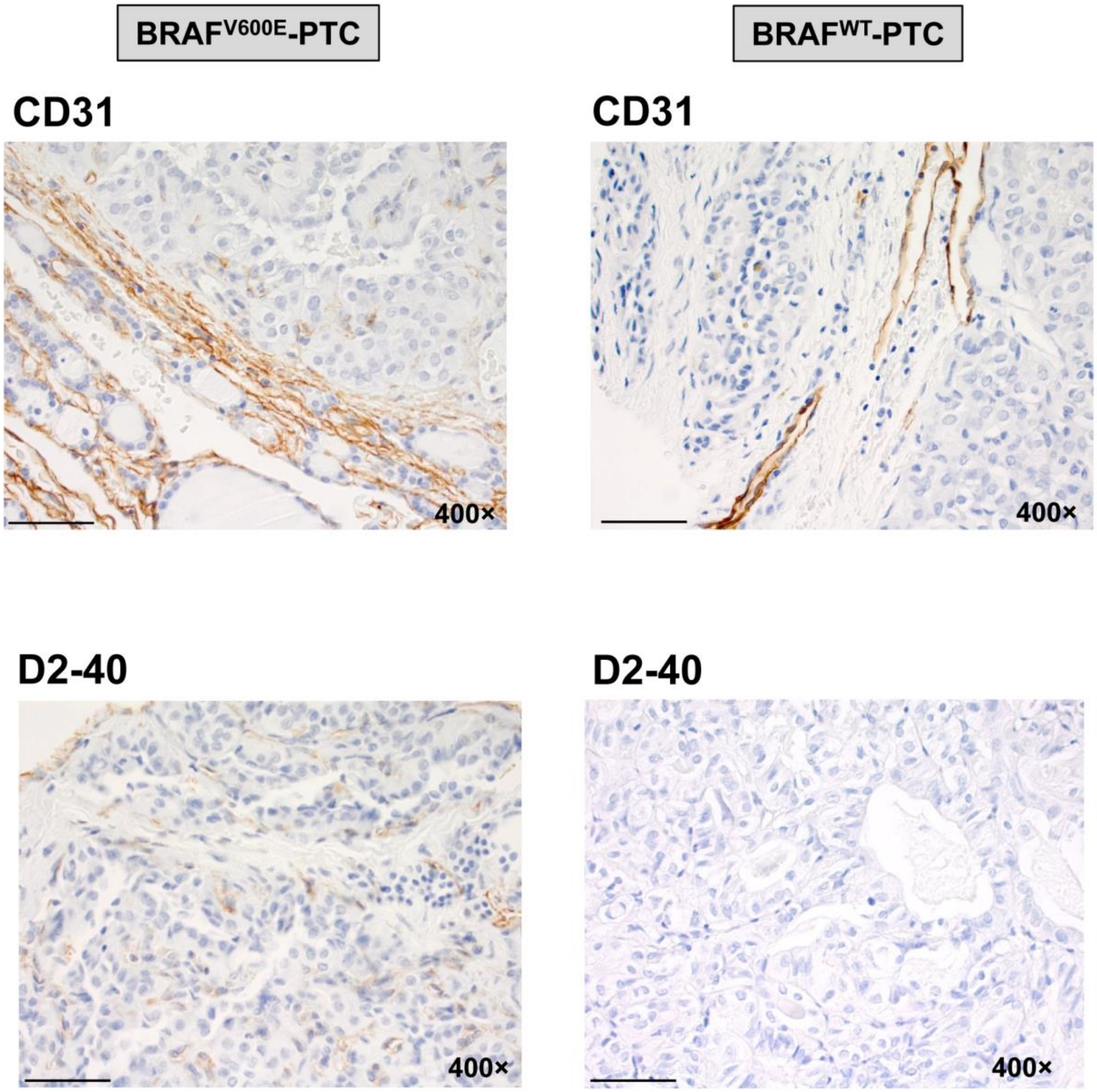

Suppl. Figure 5

Angiogenesis and lympho-angiogenesis assessment in human papillary thyroid carcinoma (PTC) with *BRAF*^{V600E} (n=4) or *BRAF*^{WT} (n=3).

Microvascular density is defined by number of vessels per microscope field exhibiting CD31 (BEC marker) or D2-40 (Podoplanin: microvascular lymphatic endothelial cell marker) staining. *BRAF*^{V600E}-PTC had greater peri-tumoral staining for CD31 and D2-40 compared with intra-tumor CD31 or D2-40 staining. *BRAF*^{V600E}-PTC had greater CD31 and D-20 protein expression levels compared with *BRAF*^{WT}-PTC in both peri-tumor and intra-tumor area. All scale bars are=400 µ.

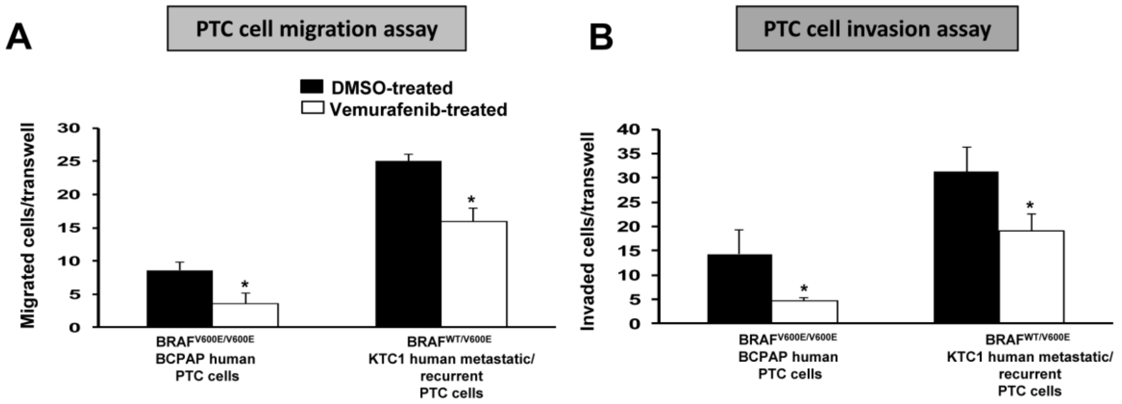

Suppl. Figure 6

Spontaneously immortalized human papillary thyroid carcinoma (PTC)-derived cell migration and invasion assays in the presence of vemurafenib or vehicle treatment.

A-B) Migration and invasion assays with vemurafenib or vehicle (control) treatment in KTC1 cells (spontaneously immortalized metastatic $BRAF^{WT/V600E}$-PTC cells and homozygous loss of *P16*) or in spontaneously immortalized non-metastatic $BRAF^{V600E/V600E}$-BCPAP cells. Treatment with vemurafenib 10 µM for migration and at invasion assays was performed. These data represent the average ± standard deviation (error bars) of 2 or 3 independent experiments replicate measurements (*$p<0.05$, Mann-Whitney test).

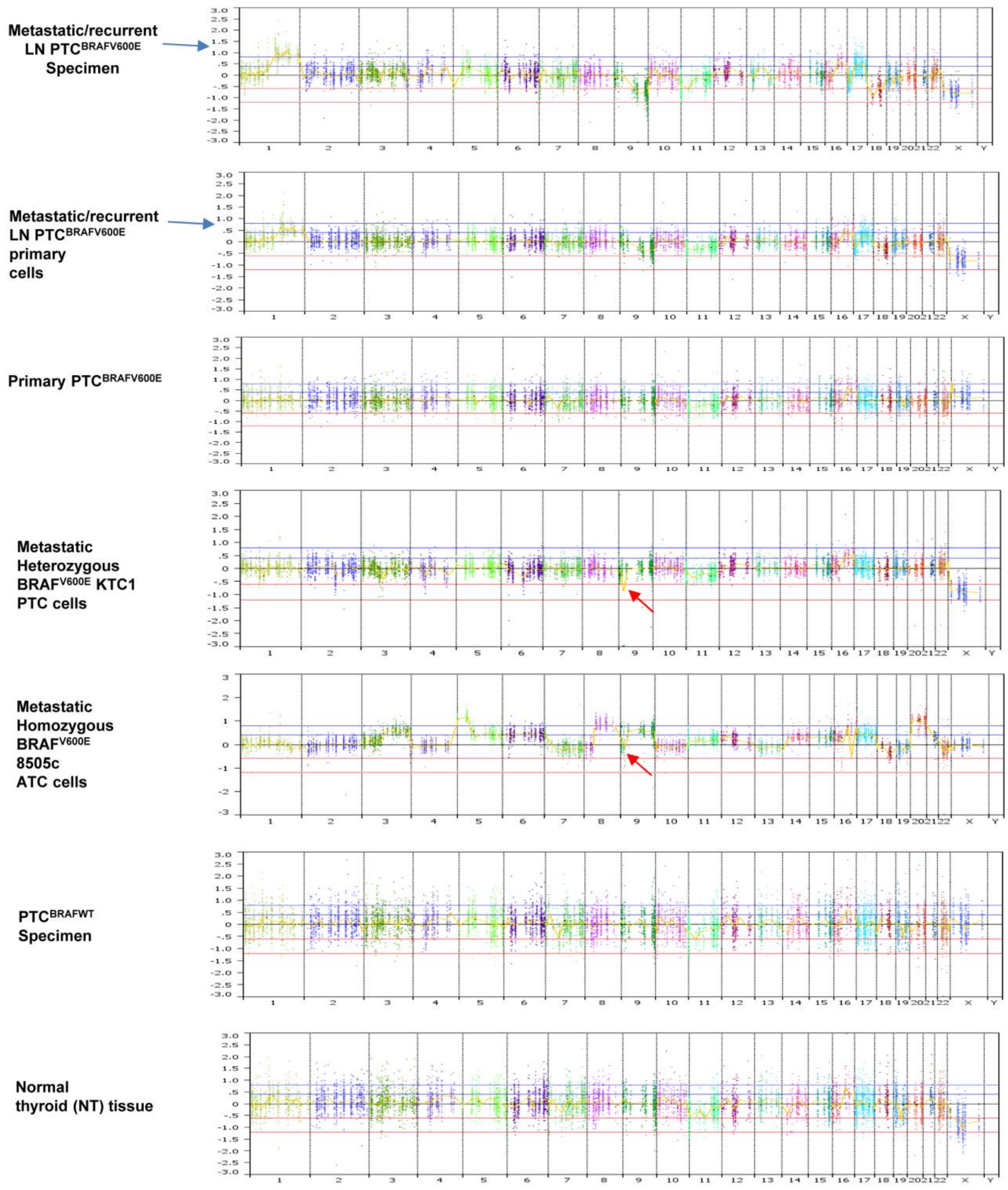

Suppl. Figure 7

Analysis of somatic copy number alterations (SCNAs) in the chromosome 1q (e.g., *MCL1 copy number gain/*amplification) or in the chromosome 9p (i.e. loss of *P16*) in human non-metastatic or metastatic thyroid carcinoma samples or in normal thyroid (NT) tissues.

Graphical representation of the log2Ratio, of sequence coverage in tumor versus reference for sequenced fragments captured by Oncopanel_v2, plotted on its genomic position. The blue and red arrows in the graphs point to the blue and red lines representing the significance thresholds for gains and losses, respectively. SCNAs were called with the following next generation sequencing (NGS) settings: significant threshold = 1E-6; max contiguous probe spacing or 1000 Kbp; minimum number of probes per segment = 3. SCNA gains has a log ration >0.4 and were called high gain if >0.8. Single copy loss threshold was -0.6 and big loss was -1.2. SCNA's on chromosomes X and Y were not called.

Suppl. Table 3

Somatic copy number alterations in 26 genes on chromosome 1q in PTC

Gene Symbols	Chromosome Region	Length	Cytoband	Probe Median	Call P-Value
MCL1, ENSA, GOLPH3L, HORMAD1, CTSS, CTSK, ARNT, SETDB1, CERS2, ANXA9, FAM63A, PRUNE, BNIPL, C1orf56, CDC42SE1, MLLT11	chr1:150,549,840-151,039,973	490134	q21.3	1.2	3.19E-102
FCRL4	chr1:162,688,854-162,730,385	19289	q23.1	1	1.54E-21
DR2	chr1:162,688,854-162,730,385	41532	q23.3	0.98	2.91E-13
ABL2	chr1:179,090,069-179,198,532	108464	q25.2	0.94	3.02E-13
MIR1278, B3GALT2, CDC73	chr1:193,091,331-193,219,842	128512	q31.2	1.05	1.17E-51
MDM4	chr1:204,494,647-204,527,809	33163	q32.1	1.08	7.06E-32
IKBKE	chr1:206,646,571-206,669,478	22908	q32.1	0.8	6.21E-36
SDCCAG8, AKT3	chr1:243,663,045-244,006,472	343428	q43 - q44	0.9	1.56E-33

Suppl. Table 4

mRNA expression levels of genes which might play a role in the biology of PTC

	Short-term primary human PTC and NT cells								Spontaneously immortalized human PTC-derived cells			
Gene	mRNA copy number (copies/10^6 18S)								mRNA copy number (copies/10^6 18S)			
Pro-angiogenic:	Metastatic/ recurrent PTC cells heterozygous $BRAF^{WT/V600E}$		Non-metastatic PTC cells heterozygous $BRAF^{WT/V600E}$		PTC cells $BRAF^{WT/WT}$		NT cells $BRAF^{WT/WT}$		Metastatic/ recurrent KTC1 PTC cells heterozygous $BRAF^{WT/V600E}$		BCPAP PTC cells homozygous $BRAF^{V600E}$	
	vehicle	vemurafenib	vehicle	vemurafenib	vehicle	vemurafenib	vehicle	vemurafenib	vehicle	vemurafenib	vehicle	vemurafenib
VEGFA	*5.8	2.3	*2.6	1.7	1.86	1.88	1.26	1.25	*4.6	2.6	*3.1	1.4
VEGFC	*4.97	1.2	5.2	5.8	2.4	2.45	2	1.3	*7.8	2.9	*1.6	0.59
VEGFR2	*0.54	0.05	*0.3	0.001	0.2	0.3	0.1	0.1	*0.4	0.02	*0.6	0.0058
NRP1	*5.5	3.3	*13.4	6.6	16.1	16.2	2.8	2.7	*8.2	0.8	*0.8	0.1
PDGFRB	*12	6.2	*4.9	2.4	3.5	3.6	1.1	1.3	*0.7	0.1	*2.3	0.5
HIF1α	*11.9	6.8	*44	22.7	42.4	45.8	7.2	7.6	*14.2	7	*25.9	15
FGF2	*21.6	7.9	*9.6	5.8	8.4	7.4	6	7.3	*9.1	4.5	*8.6	4.9
CTSB	*25.9	16.5	*23.1	13.6	19.3	19.6	16.8	18.7	*31.8	23.7	*18.7	9.6
Leptin	5.6	*7.2	4.7	4.7	3.6	3.7	2	2.1	5	*10	4.1	6
ECM remodeling:												
TSP-1	*37.2	22.1	*25.2	17.6	20.4	20.2	21.1	19.5	*0.9	0.01	*25.1	17.2
FN	*281.4	192.8	*280.2	149.1	145.3	146.5	51.4	54	*128.3	78.5	*5.3	1.1
ITGα3 (CD49C)	*25.9	20.5	*26.4	15.1	19	16.5	17.4	16	*31.5	24.4	*30.3	20.6
ITGβ1 (CD29)	*129.2	88.9	*123	58	118	116	13.8	14.5	*23.8	16.5	*38	18.8
HMGB1	*67.9	55.4	*44	22.8	31	27.2	16.1	18.9	*11.8	8.5	22.4	17.2

*$p<0.05$ ECM= extracellular matrix

Supplementary Table 5 Antibodies list and experimental conditions

Primary Antibody	Company and cat.#	Dilution and time of incubation (All antibodies were incubated for 45 minutes at room temperature except for some ones with overnight incubation at +4 degree)	Antigen retrieval (time: 30-45 minutes)
**Phospho-AKT**	Cell Signaling<br><br>4060	1:10 or 1:30, overnight	Citrate buffer and pressure cooker
**Cyclin E1**	Santa Cruz Biotechnology SC-481	1:20	Citrate buffer and pressure cooker
**Cyclin E2**	ABcam Ab32103	1:50	Citrate buffer and pressure cooker
**Cyclin B2**	ABcam Ab18250	1:75	Citrate buffer and pressure cooker
**P27**	Ab32034	1:75	Citrate buffer and pressure cooker
**Cleaved Caspase 3**	Cell Signaling<br><br>9664	1:300, overnight	Citrate buffer and pressure cooker
**MCL1**	ABcam<br><br>Ab32087	1:200	Citrate buffer and pressure cooker
**CD3**	DAKO<br><br>A0452	1:300	Citrate buffer and pressure cooker
**MAC-2**	Cedarlane<br><br>CLS942AP	1:20,000	Citrate buffer and pressure cooker
**F4-80**	E bioscience<br><br>14-4801-82	1:50	Citrate buffer and pressure cooker

**B220**	BD Bioscience<br>550288	1:500	Citrate buffer and pressure cooker
**OCT3/4**	Santa Cruz<br>SC-5279	1:40	Citrate buffer and pressure cooker
**CD90**	BD Bioscience<br>555593	1:500	Citrate buffer and pressure cooker
**P40**	E M D<br>PC373	1:1500	Citrate buffer and pressure cooker
**Vimentin**	DAKO<br>M7020	1:400	20 minutes proteinase digestion
**NG2**	Abcam<br>Cat# 83508	1:1000, overnight	10 minutes proteinase digestion
**PDGFRB**	Epitomics<br>1469-1	1:500, overnight	Citrate buffer and pressure cooker
**CD44**	Abcam<br>Ab16728	1:500	Citrate buffer and pressure cooker
**αSMA**	Sigma<br>A2547	1:20,000	none
**Desmin**	Sigma<br>D1033	1:5000	Citrate buffer and pressure cooker
**D2-40**	Covence<br>Sig-3730	1:100	none
**CD31**	Dako<br>M0823	1:30	15 minutes proteinase digestion
**P63**	Biocare	1:250	Citrate buffer and

	CM163B		pressure cooker
**pan-keratin (AE1, AE3)**	Dako<br>M3515	1:200	10 minutes proteinase digestion
**E-cadherin**	Dako<br>M3612	1:75	Citrate buffer and pressure cooker
**KI67**	Dako<br>M7240	1:30 (mouse orthotopic paraffin sections); 1:200 FFPE human tissue specimens	Citrate buffer and pressure cooker
**Phospho-ERK1/2**	Cell Signaling<br>9101	1:10 (mouse orthotopic paraffin sections), overnight; 1:150 FFPE human tissue specimens, overnight	Citrate buffer and pressure cooker
**LCA (CD45)**	Dako<br>M0701	1:600	Citrate buffer and pressure cooker
**CD68**	Dako<br>M0814	1:1000	10 minutes proteinase digestion
**P16**	Ventana<br>9517	1:2	Citrate buffer and pressure cooker
**P53**	Immunotech<br>1767	1:1200	Citrate buffer and pressure cooker
**Sodium-iodide symporter (NIS)**	Provided from Dr. Sabine Costagliola (Free University of Brussels)	1:10, overnight	Citrate buffer and pressure cooker
**Tg (thyroglobulin)**	Novus<br>NB100-65184	1:250	No treatment

**TPO (thyroid peroxidase)**	Sigma<br>A007987	1:25	Citrate buffer and pressure cooker
**TSH (thyroid stimulating hormone) receptor**	Sigma<br>A926689	1:50, overnight	Citrate buffer and pressure cooker
**PAX8**	Proteintech<br>10036-1-AP	1:800 or 1:1200	Citrate buffer and pressure cooker
**TTF1**	Dako<br>M3575	1:300	Citrate buffer and pressure cooker
**VEGF**	Abcam<br>Ab27620	1:2	Citrate buffer and pressure cooker
**VEGFR2**	Cell Signaling<br>2479	1:300	EDTA and pressure cooker
**GFP**	Santa Cruz Biotechnology<br>sc-8334	1:800, overnight	Citrate buffer and pressure cooker
**Beta-Galactosidase**	Abcam<br>Ab616	1:750	Citrate buffer and pressure cooker
